# Epigenetic regulation of gene expression in Chinese Hamster Ovary cells in response to the changing environment of a batch culture

**DOI:** 10.1002/bit.26891

**Published:** 2019-01-04

**Authors:** Inmaculada Hernandez, Heena Dhiman, Gerald Klanert, Vaibhav Jadhav, Norbert Auer, Michael Hanscho, Martina Baumann, Anna Esteve‐Codina, Marc Dabad, Jessica Gómez, Tyler Alioto, Angelika Merkel, Emanuele Raineri, Simon Heath, Daniel Rico, Nicole Borth

**Affiliations:** ^1^ Department of Biotechnology, University of Natural Resources and Life Sciences Vienna Vienna Austria; ^2^ Austrian Centre of Industrial Biotechnology Vienna Austria; ^3^ Institute of Cellular Medicine, Newcastle University Newcastle upon Tyne United Kingdom; ^4^ CNAG‐CRG National Centre for Genomic Analysis‐Centre for Genomic Regulation, Barcelona Institute of Science and Technology (BIST) Barcelona Spain; ^5^ Universitat Pompeu Fabra (UPF) Barcelona Spain

**Keywords:** Chinese Hamster ovary cells, dynamic gene expression, epigenetic regulation, long noncoding RNAs, transcriptome

## Abstract

The existence of dynamic cellular phenotypes in changing environmental conditions is of major interest for cell biologists who aim to understand the mechanism and sequence of regulation of gene expression. In the context of therapeutic protein production by Chinese Hamster Ovary (CHO) cells, a detailed temporal understanding of cell‐line behavior and control is necessary to achieve a more predictable and reliable process performance. Of particular interest are data on dynamic, temporally resolved transcriptional regulation of genes in response to altered substrate availability and culture conditions. In this study, the gene transcription dynamics throughout a 9‐day batch culture of CHO cells was examined by analyzing histone modifications and gene expression profiles in regular 12‐ and 24‐hr intervals, respectively. Three levels of regulation were observed: (a) the presence or absence of DNA methylation in the promoter region provides an ON/OFF switch; (b) a temporally resolved correlation is observed between the presence of active transcription‐ and promoter‐specific histone marks and the expression level of the respective genes; and (c) a major mechanism of gene regulation is identified by interaction of coding genes with long non‐coding RNA (lncRNA), as observed in the regulation of the expression level of both neighboring coding/lnc gene pairs and of gene pairs where the lncRNA is able to form RNA–DNA–DNA triplexes. Such triplex‐forming regions were predominantly found in the promoter or enhancer region of the targeted coding gene. Significantly, the coding genes with the highest degree of variation in expression during the batch culture are characterized by a larger number of possible triplex‐forming interactions with differentially expressed lncRNAs. This indicates a specific role of lncRNA‐triplexes in enabling rapid and large changes in transcription. A more comprehensive understanding of these regulatory mechanisms will provide an opportunity for new tools to control cellular behavior and to engineer enhanced phenotypes.

## 1 INTRODUCTION

Epigenetic modifications and regulations have attracted significant interest as researchers aim to identify the mechanisms that control gene expression. So far, most studies have focused on understanding ON/OFF mechanisms such as the complete silencing of one X‐chromosome (Brockdorff, 2011), the turning of developmental switches in embryogenesis (Shi & Wu, 2009), or cell differentiation along specific tissue lineages (Du et al., 2015). Less attention has been given to short‐term regulation of gene expression in response to environmental conditions where the majority of genes are not turned on or off, but regulated differentially to altered expression levels that enable cells to handle new conditions (Kuang et al., 2014; López‐Maury, Marguerat, & Bähler, 2008). Such mechanisms are likely to be different from ON/OFF mechanisms, but still interesting, specifically in the context of biopharmaceutical production processes where the physiological state of cells and their precise gene expression pattern have implications on bioprocess performance and product quality of biopharmaceutical proteins (Hsu et al., 2017; Stolfa et al., 2017; Yusufi et al., 2017).

To align gene expression with developmental or physiological needs, epigenetic regulators play a central role through short‐term (histone modifications) or long‐term (DNA methylation) modifications that bring about conformational changes in chromatin, thus activating or repressing transcription (G. Li & Reinberg, 2011; Saksouk, Simboeck, & Déjardin, 2015). DNA methylation, based on the conversion of cytosine to 5‐methylcytosine, tends to inhibit the binding of transcription factors or recruit repressor proteins at the methylated promoter region (Moore, Le, & Fan, 2013). Similarly, histone modifications, including acetylation, methylation, phosphorylation, citrullination, ubiquitination, SUMOylation, and ADP ribosylation of the histone tails at specific sites, contol gene transcription by modifying chromatin accessibility. For example, acetylation neutralizes the positive charge of lysine, thereby weakening DNA‐histone interactions and increasing DNA accessibility. Different forms of histone methylations, on the other hand, function based on the recognition of the position of methylated sites by effector molecules to activate or repress transcription (Bannister & Kouzarides, 2011). Both, thus, cooperate as regulators of gene transcription by controlling the interplay of transcription factors and chromatin modifiers on promoters and enhancers, resulting in changes in chromatin state. In addition, there has been a rapid increase in studies that describe transcriptional and posttranscriptional gene regulation by noncoding RNAs that function either independently or by interacting with other regulators (Dykes & Emanueli, 2017; Peschansky & Wahlestedt, 2014; Xu et al., 2017), and act as signals, decoys, guides, and scaffolds for chromatin modifiers (Marchese & Huarte, 2014; Wang & Chang, 2011). Together, these complex transcriptional dynamics result in defined patterns of gene expression and proteomic and metabolite profiles that determine the phenotype and cell survival. For instance, Kuang et al. (2014) highlight the precise temporal control of ribosome biogenesis ensuring the best utility of resources for an energetically demanding process by just‐in‐time supply within different phases of the yeast metabolic cycle.

Chinese Hamster Ovary (CHO) cells have been known as workhorses for the industrial production of recombinant therapeutic proteins since 1987 (Dorner, Bole, & Kaufman, 1987). Variations in cellular environment and phenotypes can bring about significant changes in cell behavior and productivity of producer cell lines (Pilbrough, Munro, & Gray, 2009). However, very little is known about the control mechanisms that enable rapid changes in response to environmental conditions and most transcriptome studies so far have been comparative, looking at the difference between two states or defined cell samples, such as high versus low producing cell lines. In our previous report on genomic and epigenetic variation in CHO cells (Feichtinger et al., 2016), the overall DNA methylation pattern of CHO cells was shown to change upon adaptation to different culture conditions, whereas it remained remarkably constant over months when the cells were maintained in the same medium. Short‐term changes in DNA methylation, as observed between exponential and stationary phase in CHO, were primarily found in regulatory regions such as enhancers. In addition, the first report of chromatin states, as defined by combinations of histone marks, was presented including a temporal pattern and its changes during a batch culture, however, without association to gene expression patterns. However, to achieve exquisite control over gene expression in bioprocessing, an in‐depth understanding of the mechanisms that regulate gene expression over time is indispensable. Therefore, we here follow up the previous report with the missing data on the transcriptome and its changes during the batch culture, with a particular focus on the correlation between gene expression and regulatory mechanisms. The resulting resource opens up possibilities both for enhanced control of cellular phenotypes during bioprocessing as well as the development of new engineering tools to manipulate cell behavior.

## 2 MATERIALS AND METHODS

### 2.1 Sample preparation and sequencing

CHO‐K1 cells were thawed Feichtinger et al., 2016) and, after 2 weeks of recovery, seeded into eight parallel shaker flasks at 2 × 10^5^ cells/ml, in working volumes of 250 ml. The total and viable cell count was analyzed twice daily with a ViCell (Beckmann Coulter). Samples for ChIP‐seq were taken every 12 hr (1 × 10^7^ viable cells for cell fixation and cell lysis; 1 × 10^6^ cells for magnetic immunoprecipitation), for RNA‐seq every 24 hr (1 × 10^6^ cells into 1 ml Trizol), and for whole‐genome bisulfite analysis at mid‐exponential and mid‐stationary phase (5 × 10^6^ cells for DNA isolation; Supporting Information Figure 1). For RNA extraction, the cells were centrifuged and lysed using TRI reagent (T9424–200 ml; Sigma‐Aldrich) following the manufacturer's instructions: phase separation was done by addition of chloroform and the aqueous phase collected. After precipitation with 2‐propanol and washing with 70% Ethanol, pellets were air‐dried and resuspended in nuclease‐free water. Libraries for RNA‐seq were prepared using NEBNext Ultra Directional RNA library prep Kits (E7420L), starting from total RNA according to the instructions and analyzed by Illumina HiSeq 2000 PE100 (pair‐end; 100 bp read length). Libraries for ChIP‐seq and bisulfite sequencing were prepared as described (Feichtinger et al., 2016).

### 2.2 RNA‐seq mapping and normalization

RNA‐seq reads were aligned with the GEMTools RNA‐seq pipeline v1.7 (Marco‐Sola, Sammeth, Guigó, & Ribeca, 2012) in three phases: mapping against the Chinese hamster genome published by Brinkrolf et al. (2013), against a reference transcriptome and a de novo transcriptome, generated from the input data to detect new junction sites. After mapping, all alignments were filtered for a minimum intron length of 20 bp, a maximum exon overlap of 5 bp, and a check against a reference annotation for consistent pairs and junctions, where both sites align to the same annotated gene. Mapping statistics and expression quantification were calculated by GEMTools “gtfcount,” and expressed genes were identified based on fragments/counts per million mapped fragments (FPM/CPM), filtering for rowSums >1 using the DESeq.2 R package bioconductor‐3.4.1 (Love, Huber, & Anders, 2014). Differential expression was analyzed by normalizing the raw read count of all time points (TP) with the library size.

### 2.3 New reference gene model built for coding and noncoding transcribed regions

In view of the draft state of the Chinese Hamster reference genome and the incomplete annotation of noncoding RNAs, an extended reference gene model was built (Supporting Information File), resulting in 25,541 genes with functional annotation (Supporting Information Tables 1 and 2) and 78,873 noncoding transcribed regions encoding for 80,973 transcripts, including 51,193 long noncoding RNAs (lncRNAs) or processed transcripts. Based on the presence of active chromatin marks and length distribution, 1,528 noncoding genes and 947 protein‐coding genes were annotated with an unknown function (Supporting Information Table 3).

### 2.4 Differential gene expression

The design formula for the samples was created based on the PC analysis that separated samples from different TP corresponding to the gene expression values (Supporting Information Figure 2) considering TP1, TP3, TP5, and TP7 (17–90 hr) as the exponential phase; TP9 and TP11 (114–138 hr) as the stationary phase; and TP13, TP15, and TP17 (162–210 hr) as the decline phase. Phase‐wise comparison was done between exponential and stationary (ES) and exponential and decline (ED) phases. Differentially expressed genes were extracted based on DESeq.2 normalized read counts by the Benjamini–Hochberg method to adjust *p* values with a threshold of 0.01 and an absolute value of log2 fold change >1.

Gene set enrichment analysis (GSEA) allows computation of statistical significance of predefined gene sets that share common biological function, based on a ranked list of differentially expressed genes observed while comparing two distinct states or phenotypes. This was performed with the GSEA software (v2.2.4) (Subramanian et al., 2005) based on DESeq.2 stat (Wald statistic) prerank gene‐list (Mootha et al., 2003) for phase‐wise comparison analysis. Negative phenotype corresponds to exponential phase, positive phenotype to the stationary phase in the ES comparison and the decline phase in the ED comparison. The differential expression (DE) analysis was done separately for coding and noncoding transcribed regions. All coding genes reported as DE from both phase‐wise comparisons were selected for Fuzzy clustering and Gene Ontology (GO) enrichment analysis. Gene expression matrix was normalized with the variance stabilizing transformation (VST) followed by standardization by a gene with *z*‐score normalization (*x*−mean/standard deviation) using the R clusterSim package v0.45–2 (M. E. Futschik & Kumar, 2017; Lemay et al., 2013). Clustering was performed with the Mfuzz Bioconductor package v2.36.0 (Matthias Futschik, 2017) to report four clusters (Supporting Information Figure 3). GO enrichment analysis was done with topGO R package v2.24.0 (Adrian Alexa, 2017) for genes with membership higher than 0.5 in any of the clusters.

### 2.5 DNA methylation around transcription start site

The whole genome bisulfite analysis data published in our previous report were utilized for this analysis (Feichtinger et al., 2016). The mean of methylation percentage per CpG was plotted against its distance from the transcription start site (TSS), filtering CpGs with less than 10 reads. DNA methylation upstream and downstream of TSS were assessed for expressed and non‐expressed genes, and for expressed genes containing active promoter states (states 9 and 10). For coding genes, 3 kb upstream and downstream of TSS was analyzed. For noncoding RNAs, considering their shorter length, this was reduced to 1.5 kb to avoid noise (Supporting Information Figure 4). The average CpG methylation per position was calculated and fitted by LOESS smoothing using the default span value of 0.75 with the stats package from R(v3.3.1).

### 2.6 Chromatin state enrichment

As published in Feichtinger et al. (2016), the presence and combination of 6 histone modification marks (H3K4me1, H3K4me3, H3K9me3, H3K27me3, H3K36me3, and H3K27ac) can be used to define a chromatin state model (Ernst & Kellis, 2012) that identifies genomic regions with deduced promoter, enhancer, repressor, heterochromatin, and actively transcribed region functionality. Using the overlap enrichment function of ChromHMMv1.12 (Ernst & Kellis, 2012) based on segmentation with the published 11 states model, the enrichment of each state was computed for a set of external coordinates: differentially methylated 1 Kb regions between exponential and stationary phase within batch culture, the genomic region between TSS and transcription end site (TES) for expressed, and non‐expressed coding genes as well as non‐coding RNAs.

### 2.7 Temporal association of gene expression with chromatin marks

Genes with active transcription mark (State 4) within their gene body were identified, and the corresponding coordinates were intersected for the presence of H3K36me3 peak coordinates as identified from the MACS2 peak caller (Zhang et al., 2008). Genes containing State 4 and an H3K36me3 peak within the gene body are annotated as H3K36me‐E4. Similarly, the combination of H3K4me3 peaks with states 9 or 10 (H3K4me3‐E9,10) and H3K27ac peaks (H3K27c‐E9,10) was identified around the TSS+/− 500 bp. Considering many genes of small length, a 500 bp flanking region was used to ensure capturing a pattern without interference from the neighboring genes. Changes in expression levels (*z*‐score of VST normalized values) with all active transcription marks (*z*‐score of CPM values) calculated with the DiffBind R package (Stark & Brown, 2017) were plotted in a heatmap using ggplot2 (Wickham, 2009). The Pearson correlation was calculated with the Hmisc R package version 4.0.3 and plotted with corrplot R package version 0.77 (Wei & Simko, 2017).

### 2.8 Interaction analysis for expressed lncRNA and coding genes

Sequences for all expressed lncRNA and DNA sequences of all coding genes plus 1.5 kb upstream and downstream of the gene body were extracted from the genome using samtools version‐1.3.1 (H. Li et al., 2009). Triplex‐forming oligos in lncRNA transcripts and the corresponding triplex target sites (TTSs) within and around coding genes were identified using triplexator 1.3.2 (Buske, Bauer, Mattick, & Bailey, 2012). Interactions were filtered for the presence of purine motifs with a minimum triplex length of 20 and minimum G proportion of 50%. An error rate of up to 20% and only two consecutive errors without low complexity filtering were allowed (Buske et al., 2012). The output was parsed to extract TTSs coordinates and unique pairs of interacting lncRNA and coding genes.

#### 2.8.1 Temporal association of expression levels

Changes in the expression levels of the coding genes in the neighborhood of DE lncRNAs and triplex‐forming lncRNAs were studied throughout the batch by identifying all expressed lncRNAs and their TTSs located within 1.5 kb upstream or downstream of coding genes and analyzing the probable regulation. Unique interacting lncRNA‐coding gene pairs were identified and FPM values for lncRNA and coding genes at all TPs normalized for *z*‐score within each gene pair. Trends in the changing expression of each gene are plotted in pair‐wise heatmaps with the normalized values using the ggplot2 R package (Wickham, 2009, p. 2), for both neighboring and triplex‐forming gene pairs individually, separated according to cluster classification of lncRNAs.

To demonstrate the involvement of lncRNAs in the regulation of the coding gene expression, the level of triplex‐mediated lncRNA interactions with DE coding genes was compared with that of nondifferentially expressed (NDE) coding genes. The coordinates of TTSs were extracted and overlapping coordinates merged to avoid redundancy. The percentage length covered by TTSs within and around 500 DE coding genes with maximum fold change and NDE genes with minimum fold change was plotted in Violin plots showing the distribution of the percentage with the probability density of data. The correlation coefficient for changing expression levels across all TPs was calculated for the interacting gene pairs based on the *z*‐score normalized FPM values and filtered for interactions with only DE lncRNAs.

#### 2.8.2 Localization of interactions

The enrichment of TTSs was analyzed within the published chromatin states (Feichtinger et al., 2016). The 11 states were merged to repressed states (states 1 and 3), enhancer states (5, 6, 7, and 8), promoter states (9, 10, and 11), quiescent (2), and active transcription state (4). The lengths of all TTSs (from the nonredundant merged coordinates) in each state were summed and normalized by the total length of state within the gene regions under consideration (1.5 kb upstream and downstream of gene body). The normalized frequencies, ratios of the total count within a chromatin state, and total length occupied by this state were plotted for all 18 TPs.

### 2.9 Quantitative polymerase chain reaction validation of DE

Isolated RNA (800 ng) was reverse‐transcribed with the High‐Capacity cDNA Reverse Transcription Kit (Thermo Fisher Scientific) including an RNase inhibitor. For each sample, a reverse transcription (RT) control was included, which was treated equally, but without the addition of reverse transcriptase. The generated complementary DNA samples and RT controls were 1:4 diluted with nuclease‐free water and analyzed in quadruplet reverse transcription polymerase chain reaction (RT‐PCR) reactions of 10 µl with SensiFAST^TM^ SYBR® Hi‐ROX Kit (Meridian Bioscience). qPCR was performed on a Rotor‐Gene Q (Qiagen, the Netherlands) and transcript levels determined by the $2^{-\Delta C_{t}}$ method against housekeeping genes PLEKHA5 (cgriseus1B003354) and CUL7 (cgriseus1B027447; Livak & Schmittgen, 2001).

## 3 RESULTS

### 3.1 Transcriptome response of CHO cells during batch culture

Batch culture is a perfect example for changing conditions, with significant environmental variation and altered media composition during different growth phases (Young, 2013). To address the phenotype‐relevant changes in gene expression, a high‐resolution temporal profile of global gene transcription for CHO‐K1 cells was analyzed by RNA‐seq every 24 hr over 9 days (Supporting Information Figure 1) and related to previously published changes in the chromatin state and the global DNA methylation pattern (Feichtinger et al., 2016).

### 3.1.1 DE during growth phases

To explore the similarity of the transcriptomes analyzed at different time‐points, a principal component (PC) analysis was performed over the expression levels of all expressed coding and noncoding transcribed regions (Supporting Information Figure 2, Supporting Information Table 4). A cumulative percentage variance of 94.2% was accounted for by PC1. No separation across PC1 shows the overall similarity across all samples. This is not surprising, as 96% of all expressed genes are constantly expressed. However, PC2, accounting for 1.9% of the variability, separates samples into three different growth phases: exponential, stationary, and decline. We found 14,547 protein‐coding genes to be expressed (rowSums >1), of which 188 genes were DE (false discovery rate [FDR] < 0.01) between exponential and stationary phase (ES) and 1,381 between exponential and decline (ED) phase (Supporting Information Table 5). In total, 1,397 unique coding genes show DE between growth phases, comparable with a previous study (Bort et al., 2012). Gene expression during exponential growth remains surprisingly stable, despite the already changing environment. During transition into stationary and decline phase, however, the pattern begins to change.

Using GSEA with default parameters (FDR < 0.25) (GSEA/MSigDB Team, 2018), 224 gene sets were identified to be enriched in exponential phase relative to stationary phase (ES; Supporting Information Table 6a), including growth enhancing pathways such as glucose transport, cancer‐related, and cell proliferation pathways, such as TNFR2 and Myc pathways (Martinato, Cesaroni, Amati, & Guccione, 2008; Slavov, Budnik, Schwab, Airoldi, & van Oudenaarden, 2014; Young, 2013). The Myc pathway constitutes a clear example of growth regulation and differentiation through chromatin state modification. It binds to target promoters, modifying chromatin states through the promotion of hyperacetylation in multiple lysines and contributing to the regulation of transcription (Martinato et al., 2008). In contrast, 44 gene sets were identified to be enriched in stationary phase relative to exponential phase, including pathways related to protein degradation and nitrogen metabolism, lysosome, cell binding, cell signaling, and remodeling of extracellular matrix. At this stage, cellular homeostasis and housekeeping processes appear to be enriched, to ensure prolonged viability in response to altered media composition and lack of nutrients. Finally, comparing exponential to decline phase (ED), 235 gene sets showed enrichment in exponential phase and 95 gene sets in the decline phase (Supporting Information Table 6b). Several gene sets enriched in exponential phase are related to DNA damage and genomic instability, which are known to be highly prevalent in rapidly growing cells, such as CHO. The results suggest that diverse mechanisms for DNA repair and stress response decrease in the decline phase, possibly leading to a higher rate of genome damage (Bort et al., 2012). For instance, pathways related to cell cycle checkpoints appear in the top 20 significant pathways enriched in ED. Interestingly, lipid metabolism is a major response factor during the decline phase, indicating the cells' need to activate energy resources. Table 1 shows a subset of pathways enriched in the different growth phases (details in SuppTable 6).

**Table 1:** Subset of most significantly enriched gene sets in different growth phases. The table enlists overrepresented gene sets detailing the number of genes in the gene sets being used from the input data (size) with other GSEA statistics including enrichment score (ES), normalized enrichment score (NES), nominal *p* value and false discovery rate (FDR)

	Gene set name	Database	Size	ES	NES	NOM *p* value	FDR *q* value
Exponential vs. stationary phase	Gene sets overrepresented in exponential phase						
	Tumor necrosis factor	ST	26	−0.70	−2.19	0	1.21E−04
	Myc active	PID	62	−0.59	−2.28	0	0
	TNFR2	Biocarta	16	−0.67	−1.89	0	0.01
	DNA replication	KEGG	20	−0.63	−1.85	0.004	0.02
	Glucose transport	Reactome	31	−0.57	−1.87	0.002	0.01
	Gene sets overrepresented in stationary phase						
	Lysosome	KEGG	100	0.62	2.58	0	0
	Extracellular matrix organization	Reactome	52	0.51	1.85	0	0.04
	Glycosaminoglycan degradation	KEGG	16	0.70	1.94	0.004	0.02
	Glycosphingolipid metabolism	Reactome	29	0.56	1.81	0.002	0.045
	Galactose Metabolism	KEGG	19	0.58	1.69	0.008	0.10
Exponential vs. decline phase	Gene sets overrepresented in exponential phase						
	DNA replication	Reactome	87	−0.71	−2.63	0	0
	Cell cycle	Reactome	203	−0.63	−2.62	0	0
	ATR in response to replication stress	Reactome	34	−0.76	−2.43	0	0
	Cell cycle checkpoints	Reactome	56	−0.71	−2.47	0	0
	Homologous recombination	KEGG	21	−0.79	−2.20	0	0
	Gene sets overrepresented in decline phase						
	Extracellular Matrix Regulators	NABA	125	0.64	2.75	0	0
	Lysosome	KEGG	100	0.64	2.62	0	0
	Integrin1	PID	52	0.69	2.59	0	0
	Collagen formation	Reactome	38	0.67	2.34	0	0
	Lipid digestion, mobilization and transport	Reactome	25	0.64	1.98	0	0.006

### 3.1.2 Gene expression clusters

**Figure 1:**
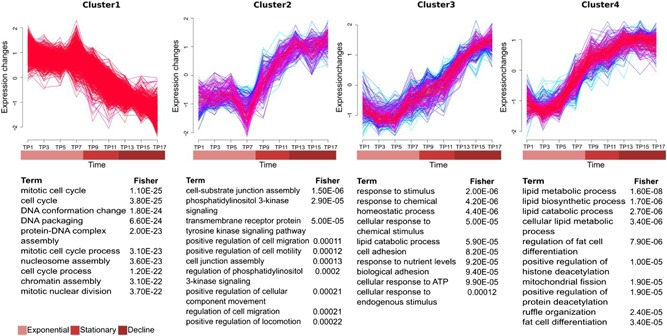
Expression patterns of differentially expressed protein‐coding gene clusters during batch culture of CHO cells. The *y*‐axis represents the *z*‐score (number of SDEV from the mean). Line colors indicate membership values of each gene ranging from blue (for low membership <0.5) to red (for high membership >0.5). The top gene ontology terms enriched are tabulated under each cluster with classic Fisher values. CHO: Chinese Hamster Ovary [Color figure can be viewed at wileyonlinelibrary.com]

A soft clustering algorithm from the mfuzz R package revealed four different gene expression profiles for 1,397 DE protein‐coding genes. PC analysis separated expression profiles during batch culture into two main groups ‐ Cluster 1 and Cluster 2, 3, and 4 (Figure 1; Supporting Information Figure 3, Supporting Information Table 7). The biological role of each cluster of genes was determined by GO enrichment (Supporting Information Table 8). Cluster 1 is gradually decreasing in expression from exponential to decline phase, with 706 coding genes. As expected, the majority of these are related to mitotic cell cycle, chromatin organization, DNA damage/repair, and RNA biogenesis, all major prerequisites for growth and proliferation. Clusters 2 (188 genes), 3 (242 genes), and 4 (261 genes) increase in expression levels from exponential to decline phase, in different patterns. GO annotation confirms the result from GSEA as the majority of these upregulated pathways were related to lipid metabolism, cell homeostasis, cell motility, and extracellular matrix organization (Supporting Information Table 9). Each cluster was validated for their temporal expression profile by qRT‐PCR of a selected number of genes (Supporting Information Figure 5, Supporting Information Table 10).

### 3.2 Mechanism of regulation of coding genes in response to culture conditions

To understand the mechanism underlying this response in the gene expression pattern, changes in the major epigenetic regulators including DNA methylation and histone modification (Kundaje et al., 2015) were interrogated.

#### 3.2.1 DNA methylation

Various genome‐wide studies have established the role of DNA methylation in control of gene expression, according to the location within the transcriptional unit. Demethylated promoters and methylated gene body have been reported to promote active transcription (Huang et al., 2015; Jones, 2012), although after nucleosome assembly, transcription cannot be initiated for genes with methylated CpGs around the TSS (Hashimshony, Zhang, Keshet, Bustin, & Cedar, 2003; Jones, 2012; Kass, Landsberger, & Wolffe, 1997). For our data, the DNA methylation pattern was found to be clearly distinct around TSS for expressed and nonexpressed genes. As expected (Huang et al., 2015), we observed extremely low methylation levels for expressed and complete methylation for nonexpressed genes (Supporting Information Figure 6). Consistent with previous reports (Kundaje et al., 2015; Sharp et al., 2011), the methylation level is noticeably lower around TSS for genes also bearing active promoter marks for both coding and noncoding transcribed regions (Supporting Information Figure 6b). Importantly, modifications of the global DNA methylation pattern between exponential and stationary phase were observed mostly in genomic regions with regulatory chromatin states, rather than promoter marks (Feichtinger et al., 2016), indicating that while DNA methylation in promoters marks gene expression as “ON” or “OFF,” it is not the rapid response mechanism for fine‐tuned control of expression level such as is required for short‐term response during a batch culture.

#### 3.2.2 Chromatin modifications

**Figure 2:**
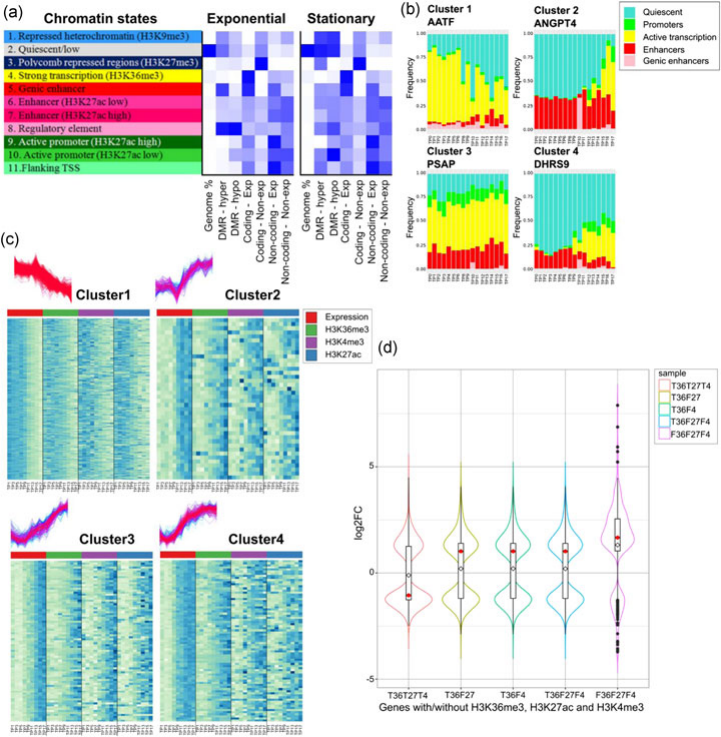
Regulation of gene expression and histone modifications. (a) Distinct enrichment of chromatin states within expressed and nonexpressed transcribed regions and differentially methylated regions (DMRs; chromatin states as determined in (Feichtinger et al., 2016)): between exponential and stationary growth, well‐defined shifts in enrichment of active (states 4 and 5) and repressive (states 1, 2, and 3) chromatin states within expressed and nonexpressed genes, respectively. Interestingly, enrichment of DMRs shifts from the genic enhancers in the exponential phase to the repressive states in stationary phase. (b) Changing enrichment of chromatin states with gene expression throughout the batch culture on the example of one gene (TSS–TES) from each cluster of the DE coding genes. Changes in the levels of enrichment of chromatin states (particularly active transcription state: yellow) follow the same trend as the expression in the cluster to which the gene belongs. (c) Temporal association of gene expression with individual chromatin marks— H3K36me3, H3K4me3, and H3K27ac for DE coding genes. Levels of individual active chromatin marks (green, violet, and blue) also show the same trend as transcript expression levels (red). (d) Distribution of fold changes of DE genes that bear individual active chromatin marks—H3K36me3, H3K4me3, and H3K27ac or combinations thereof (Unfilled black dot marks–distribution mean; filled red dot marks–distribution median). Fold change distribution for genes carrying all three active histone marks is towards negative, depicting higher expression in the exponential stage, while for genes carrying combinations of two marks, the distribution median is towards positive, indicating upregulation in late culture stages. However, the genes carrying none of these histone marks, at any time point throughout the batch, seem to have the highest fold change distribution, with a tendency towards upregulation in late culture phases. DE: differential expression; TES: transcription end site; TSS: transcription start site [Color figure can be viewed at wileyonlinelibrary.com]

Many studies confirm that alterations in histone modifications lead to changes in chromatin conformation that control gene expression as and when required (Kuang et al., 2014; López‐Maury et al., 2008). The PC analysis of histone modifications revealed their continuous adaptation (Feichtinger et al., 2016), even during exponential phase where gene expression patterns are very uniform (Figure 1). The 11 chromatin states computed from 6 histone marks (Feichtinger et al., 2016) allow us to identify the function of genomic regions of concern. Various categories of genomic regions with different features were checked for enrichment in these chromatin states. Coordinates for coding genes were extracted from 2 kb upstream of the TSS to TES and from only the gene body for noncoding RNAs. For nonexpressed coding genes, Figure 2a shows high enrichment of repressive and quiescent chromatin states (H3K9me3 State 1, H3K27me3 State 3, and quiescent State 2). In contrast, these marks are absent for expressed genes which are instead enriched for active transcription and genic enhancer states (states 4 and 5). In addition, enrichment of chromatin states within differentially methylated regions could help in understanding the underlying regulatory mechanisms that enable cells to respond rapidly to environmental conditions. Interestingly, in the exponential phase, hypermethylated regions are enriched within genic enhancers, indicating high activity in expression (actively transcribed regions need to be fully methylated, unlike promoters that need to be demethylated to be active). In stationary phase, on the other hand, they are enriched within Polycomb repressed regions. Also, the differentially methylated regions (either hypomethylated or hypermethylated) were found to be enriched within regulatory elements (chromatin states 5–8) during exponential phase, which moved to quiescent and repressed regions (chromatin states 1–3) during stationary phase (Schröder et al., 2017).

#### 3.2.3 Temporal changes of histone modifications

All DE coding genes were checked for the presence of H3K36me3‐E4 within the gene body and for H3K4me3‐E9,10 and H3K27ac ‐E9,10 peaks around the TSS. Supporting Information Figure 7a shows significant (*p* < 0.01) positive correlation between changes in expression levels and histone modifications for the three active histone marks, especially in early exponential and decline phases. Figure 2b,c show continuous adaptation of chromatin modifications with differential gene expression in the coding gene clusters. The highest significant (*p* < 0.01) correlation is observed for H3K36me3‐E4 marks (Supporting Information Figure 7a). Read distribution for hyperacetylation of different phase‐wise comparisons (ES, ED, and SD) shows that the genome has more hyperacetylated sites in exponential phase (Supporting Information Figure 8), which agrees well with the higher transcriptional changes in this phase (Schröder et al., 2017; Sharp et al., 2011). Moreover, out of 1,397 DE coding genes, 986 genes had H3K36me3‐E4 within their gene body, but only 731 had H3K4me3‐E9,10 and 721 had H3K27ac‐E9,10 around the TSS, and only 647 genes were found to have all three active chromatin marks. Genes that fall into these groups (all histone marks present, only two, only one, and none) have distinctly different patterns of regulation over the culture (Figure 2d): those genes that bear all three histone marks mostly decrease expression level towards the end of the batch, those that bear only one or two respective marks are modestly upregulated and, most interestingly, those that bear no histone marks have the highest fold changes.

#### 3.3 Long noncoding transcripts and their potential function in rapid response

**Figure 3:**
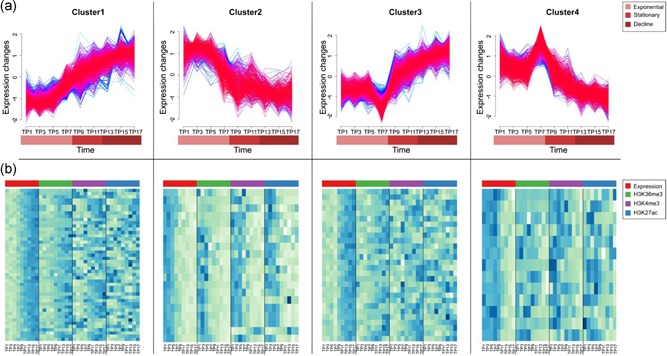
Expression patterns of differentially expressed noncoding RNA clusters during batch culture of CHO cells. (a) Clusters of differentially expressed noncoding RNAs during batch culture of CHO cells. (b) Temporal association of expression levels with chromatin marks—H3K36me3, H3K4me3, and H3K27ac for DE lncRNAs. Similar to Figure 2c for coding genes, the levels of active histone marks follow the same trend as expression levels of noncoding RNAs. CHO: Chinese Hamster Ovary; DE: differential expression; lncRNAs: long noncoding RNAs [Color figure can be viewed at wileyonlinelibrary.com]

As per the deduced annotation from the transcript assembly using the RNA‐Seq data, 74% of the annotated Chinese Hamster genes encode for noncoding RNAs, 62% of these are lncRNAs or processed transcripts. Around three times more noncoding transcribed genes (42,177) in comparison to protein‐coding ones were found to be expressed (rowSums >1). Such a high number suggests an important role in regulation. However, overall expression levels of noncoding RNAs were found to be much lower than that of protein‐coding genes (Supporting Information Figure 9). All expressed lncRNA genes were checked for homology against the 279 lncRNAs reported with known function in lncRNAdb, using default parameters from BLAST. We found 2,565 lncRNAs to have homology with only 56 lncRNAs in lncRNAdb, with alignment length ranging from 28 to 4,049 nucleotides and a minimum percentage identity of 71.35%. Such an overall low count of lncRNAs with known function shows the necessity of a better understanding of the functional relevance of these abundant regulators. Gene expression profiling and in situ hybridization provide various evidence that lncRNA expression is differentially regulated spatially, temporally or in response to stimuli (Derrien et al., 2012). DE analysis over the three growth phases (FDR < 0.01) reported 456 noncoding RNAs DE between exponential vs stationary phase and 2,863 DE between exponential vs decline phase (Supporting Information Table 11). In total, 2,899 unique noncoding RNAs show DE, 94% of which are lncRNAs. Clustering of DE noncoding RNAs reported four trends (Figure 3a; Supporting Information Table 12). As with the coding genes, the genomic region around TSS was found to be demethylated for expressed and methylated for silenced genes (Supporting Information Figure 6). Correlations of changes in lncRNA expression and histone modifications across the batch are presented in Figure 3b. While 583 lncRNAs had H3K36me3‐E4 within the gene body, 791 had H3K27ac‐E9,10 and 829 H3K4me3‐E9,10 around the TSS. All three active transcription marks were found only on 108 differentially expressed lncRNAs and plotted with expression levels for each cluster (Figure 3b). Although the trend of active transcription mark (H3K36me3) shows a highly significant (*p* < 0.01) correlation (Supporting Information Figure 7b) with expression levels, the trends for promoter marks (H3K4me3 and H3K27ac) behave noisy (Figure 3b; Supporting Information Figure 7b).

#### 3.3.1 DE lncRNAs and their impact on neighboring coding genes

**Figure 4:**
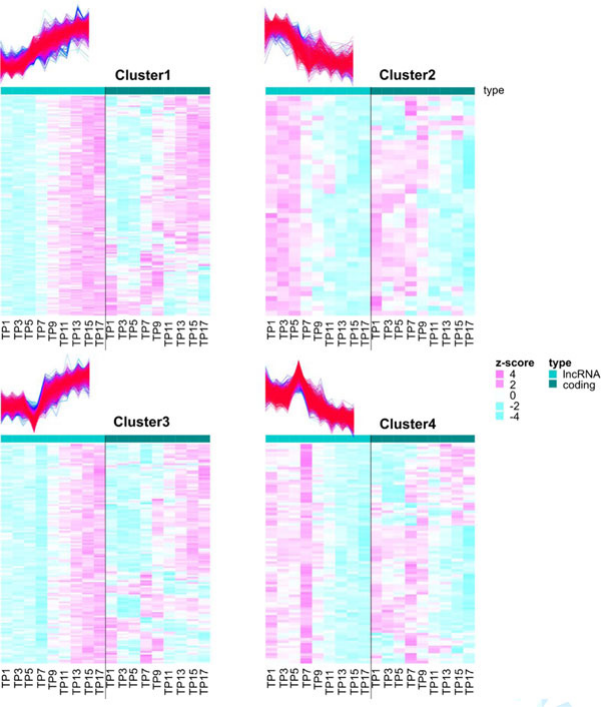
Temporal association of expression levels within neighboring lncRNA‐coding gene pairs (DE lncRNA, all coding genes). Heatmap displays trends of lncRNA expression within 1.5 kb distance upstream or downstream of a coding gene to observe the plausible role of lncRNA in regulating expression of the neighboring coding gene. Interestingly, the expression levels of coding genes (right panels) neighboring a DE lncRNA (left panels), shifts from high to low or vice‐versa around the same time as the expression of the lncRNA in either positive (plausibly enhancing transcription) or negative (plausibly repressing transcription) correlation. DE: differential expression; lncRNAs: long noncoding RNAs [Color figure can be viewed at wileyonlinelibrary.com]

To investigate the association of expression levels between pairs of lncRNAs and neighboring coding genes, expression levels were plotted for those lncRNA‐coding gene pairs where a DE lncRNA was found within 1.5 kb distance upstream or downstream of the coding gene body (Supporting Information Table 13). To analyze the trend, this list of 387 DE lncRNAs was separated according to the lncRNA clusters. As expected, Figure 4 shows that gene expressions are both positively as well as negatively correlated. The positive correlation could be due to sharing the same transcriptional machinery in neighboring chromatin domains or to a potential role of cis‐regulation of lncRNAs to the neighboring coding genes. Negative correlation, as proven in a number of studies, points towards transcriptional repression of coding genes by the expressed lncRNAs in cis. Moreover, it is interesting to note that despite selecting all 14,157 expressed coding genes, the transcriptional pattern of coding genes for each gene pair with a DE lncRNA is never stable. Instead, the expression levels for coding genes mostly changes at the same TP when lncRNA expression begins to change. Such a pattern hints towards lncRNAs being strongly involved in regulating their neighboring genes. Corresponding patterns for these pairs filtered for significantly DE coding genes are shown in Supporting Information Figure 10a.

#### 3.3.2 Interacting pairs of DE lncRNA and distant coding genes

**Figure 5:**
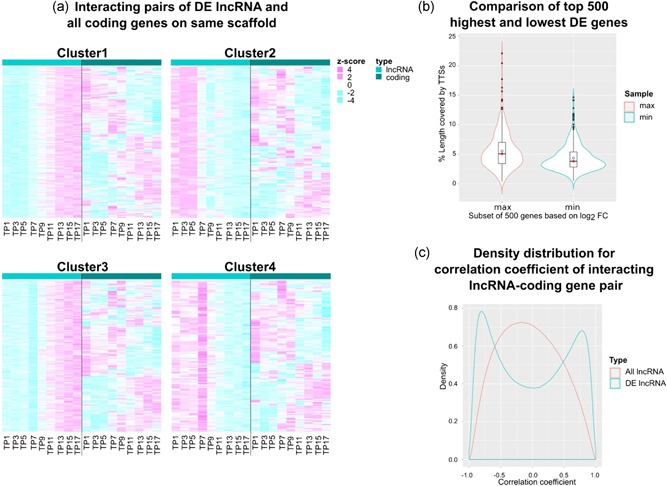
Regulation of coding gene expression by lncRNAs. (a) Correlation of expression levels within a subset of interacting lncRNA‐coding gene pairs on the same scaffolds (DE lncRNA, all coding genes). Expression levels of coding genes in triplex forming lncRNA‐coding gene pairs are never stable when the interacting lncRNA is DE. (b) Frequency distribution of percentage length covered by TTSs within 1.5 kb upstream TSS and 1.5 kb downstream TES for the 500 genes with the highest and the lowest fold change during the culture, respectively. Genes with higher log2 fold change have a higher number of triplex interactions than those with low log2 fold change. (c) Comparison of density distribution for correlation coefficient of complete interactome with all lncRNAs (red) and only DE lncRNAs (blue). The distribution peak of “All lncRNAs” (red) (i.e. DE+non‐DE) at correlation = 0.0 shows a normal distribution, whereas the expression of the majority of DE lncRNAs (at 1.0) correlates to the expression of the targeted coding genes, either positively or negatively. This observation hints towards the importance of lncRNA expression in regulating coding gene expression. DE: differential expression; lncRNA: long noncoding RNA; TES: transcription end site; TSS: transcription start site; TTSs: triplex target sites [Color figure can be viewed at wileyonlinelibrary.com]

**Figure 6:**
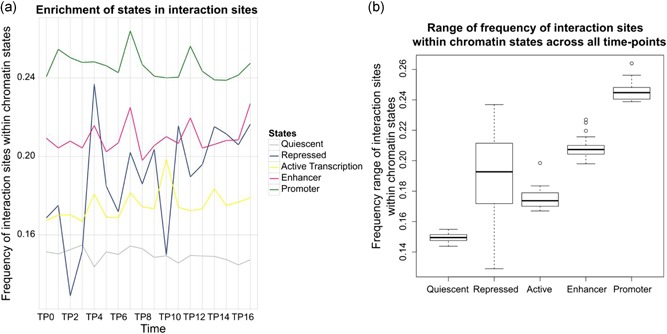
Localization of triplex interaction sites in different chromatin states. (a) Line plot reporting localization of triplex‐forming interaction sites throughout the batch culture, color‐coded for different chromatin states. (b) Boxplot showing frequency range of interaction sites within different chromatin states across all time points. The figure clearly shows enrichment of interaction sites within regulatory regions of coding genes, particularly the promoter and enhancer regions, hinting towards a regulatory function of lncRNAs in these gene pairs and their involvement in controlling the expression of the corresponding coding genes. lncRNAs: long noncoding RNAs; TP: time point [Color figure can be viewed at wileyonlinelibrary.com]

While many studies report co‐expression and localization in promoter regions as the mechanism for cis‐acting lncRNAs, a trans‐mode of action has long been known, but its mechanism is not yet well established. The homologous base pairing has been suggested as the general mechanism for posttranscriptional regulation by trans‐acting lncRNAs to gain locus specificity. Recent studies also report the presence of RNA–DNA triplex formation in the regulatory regions, and propose it as a plausible mechanism for the interaction of lncRNAs with coding genes (Jalali, Singh, Maiti, & Scaria, 2017; Mondal et al., 2015, p. 3). To investigate this, plausible triplex forming interaction sites between lncRNA transcript sequences and DNA sequences of coding genes (1.5 kb upstream and downstream TSS) were estimated using triplexator. Of 41,171 lncRNAs or processed transcripts, 24,171 were found to interact with 14,460 coding genes through 33,154,067 unique interactions in total. Frequency distribution of unique interacting targets for each such noncoding RNA showed that most noncoding RNAs have few target coding genes and few lncRNAs have many target coding genes (Supporting Information Figure 11). This information can be used to estimate essentiality of lncRNAs for cell viability based on the number of interactions (Peláez & Carthew, 2012; Reinhart et al., 2000). To verify the temporal association of expression levels between interacting gene pairs, the changes in expression values for a subset of such lncRNA‐coding gene pairs (where interacting genes are annotated on the same scaffold) were plotted side‐by‐side (Figure 5a; Supporting Information Figure 10b, Supporting Information Table 14). Most pairs were found to be either positively or negatively correlated with the trend of coding gene expression changing exactly around the time‐point for change in expression of lncRNA, as observed in the case of neighboring genes irrespective of predicted interaction (Figure 4). Interestingly, there is a bias of lncRNA interactions within the 500 genes having maximum and minimum fold change during the batch culture based on percentage length of gene covered by estimated interaction sites (Figure 5b): although there is considerable overlap, the mean of the distribution with maximum fold change was found to be significantly greater than the mean for distribution with minimum fold change. This would imply that DE coding genes with the highest rate of change in expression level over the batch culture have more triplex‐target sites around them, indicating the possible causality of high DE by triplex‐forming lncRNAs. Moreover, while the density distribution peak for the correlation coefficient of interacting lncRNA‐coding gene pairs is near 0 when all lncRNAs are taken into account, the distribution shifts to bimodal (dropping at 0) with peaks in extremes, when the list is filtered for DE lncRNAs (Figure 6c). This indicates that the probability of expecting an influence on expression levels of coding genes is higher with DE interacting lncRNAs than with NDE interacting lncRNAs. Taken together, DE of lncRNAs can be clearly associated with the temporal regulation of coding gene expression.

#### 3.3.3 Localization of interactions

The interplay of lncRNA with transcription factors and chromatin modifiers in and around the coding genes has been widely reported. Chromatin‐enriched RNAs specifically bound to regions marked with active chromatin marks (H3K4me1, H3K4me3, and H3K27ac) and RNAPII were observed by the GRID‐Seq method (X. Li et al., 2017). A genome‐wide RNA‐chromatin interactome by GRID‐Seq also reported enrichment of RNA‐interactions on active promoters and enhancers. Mondal et al. (2015) showed that MEG3 lncRNA regulates the TGF‐β pathway by RNA–DNA–DNA triplex formations. Recently Jalali et al. (2017) also reported genome‐wide enrichment of RNA–DNA–DNA TTSs in promoter regions of the human genome. To further investigate such interaction localizations in our data, enrichment of triplex‐target sites was analyzed within the previously published chromatin states (Supporting Information Table 15). As expected, it was observed that interaction sites were mostly localized within regulatory regions, especially promoter regions, rather than the highly prevalent quiescent regions or actively transcribed regions marking the gene body (Figure 6). The wide range shown by enrichment within repressor marks at different TP could be due to the fact that genome‐wide localization of these states was found in very short spans of just 200 nucleotides in most cases, which may have led to noisy results. On the other hand, the detection of triplex‐target sites within such short spans is indicative for the bias of enrichment within regulatory regions and confirms the report from Mondal et al. (2015) that describes the targeting mechanism of repressive chromatin associated lncRNAs.

#### 3.3.4 Evaluation of lncRNA gene targets

The well‐characterized lncRNAs‐MALAT1 (metastasis‐associated lung adenocarcinoma transcript 1) and NEAT1 (nuclear enriched abundant transcript 1) were selected to evaluate the gene targets reported in our interaction list reporting triplex forming lncRNA‐coding gene pairs. MALAT1 has been reported to regulate gene sets associated with cellular proliferation, localization, apoptosis, and metabolic processes and thereby plays an important role in tumorigenesis (Liu et al., 2017). It is localized to serine and arginine‐rich splicing factors (nuclear speckles). NEAT1 forms paraspeckles with its loci adjacent to MALAT1. Similar to MALAT1, NEAT1 is also reported to be a transcriptional regulator of various genes involved in cancer progression.

Homologues of MALAT1 (cgriseus1ncB038456) and NEAT1 (cgriseus1ncB038466) were highly upregulated in the later culture phases as compared with the early exponential phase. The coding gene pairs were extracted for these lncRNAs from our interaction list, and KEGG pathway enrichment was performed individually for coding genes corresponding to MALAT1 and NEAT1 homologues (Supporting Information Table 16). As shown in Supporting Information Figure 12, all the pathways enriched in our gene lists seem to be highly representative of the functional roles associated with MALAT1 and NEAT1. In addition, a recently developed technology—Capture Hybridization Analysis of RNA Targets (CHART)—was utilized by West J. A. et al to identify the genomic binding sites for NEAT1 and MALAT1 lncRNAs in human cell lines. Performing proteomic analysis over CHART‐enriched material (CHART‐MS), the authors reported proteins associated with NEAT1 and MALAT1 in vivo. Of the 885 genes reported by CHART‐MS, 727 genes were found to be expressed in our cell lines, and 65% of those were also identified in our triplex forming gene pairs corresponding to MALAT1 and NEAT1. In addition, a comprehensive review published recently describes MALAT1 as highly associated with human cancers and presents a list of 28 genes that regulate the expression of MALAT1 during transcriptional and posttranscriptional processing (Zhao et al., 2018). Amongst the listed genes, one was not found annotated for Chinese Hamster and one not expressed in our cell lines, however, 18 coding genes of the remaining were identified to have lncRNA TTSs within or around their gene body. Hence, the here reported triplex mediated interaction list could be highly useful in regulating certain pathways or gene sets of interest by controlling the expression of the associated lncRNAs.

## 4 DISCUSSION

A plethora of studies have reported dynamic changes in chromatin conformation to be associated with transcription factor binding and subsequent RNA expression (Koike et al., 2012; Kuang et al., 2014). However, there has been a dearth of high‐resolution temporal analysis correlating gene expression in response to environmental rather than developmental signals to all the major pillars of epigenetic regulation (Bar‐Joseph, Gitter, & Simon, 2012; Kundaje et al., 2015). This report presents a comprehensive high‐resolution view of transcription profile in different phases of a CHO cell batch culture along with the on‐going cross‐talk with DNA methylation, histone modifications and noncoding RNA interaction.

The dynamic response of cells to a changing environment and the continuous adaptation of their gene expression pattern are reflected in the gene expression clusters analyzed and the corresponding pathways that change as cells respond. The metabolic shift between culture phases demonstrates the dynamic response of cells and the continuous adaptation of the gene expression pattern to the changing environmental conditions (depletion of substrates and accumulation of waste metabolites). Due to the high resolution of closely spaced analysis TPs, transient changes in expression and regulatory interactions could be captured. We could show a clear association of the major epigenetic marks with the expression levels of both protein‐coding and noncoding genes. While the enrichment or depletion of DNA methylation around the TSS determines whether a gene is actively transcribed or not, functioning as an ON/OFF switch, the effect is more pronounced in the presence of active promoter states (Supporting Information Figure 6). The second major determinant for the level of gene expression was the presence of an active transcription state mark on gene bodies decorated by H3K36me3, where a strong correlation between the trend in levels of active histone marks and expression levels across different TPs of the batch culture was observed (Figure 2). Notably, for the genes with the highest fold change, the presence of activating histone marks is less pronounced but is compensated for by a higher frequency of triplex‐forming target sites of DE noncoding RNAs. These appear to enable a more rapid and more pronounced regulation of gene expression that is possibly also more short‐lived. The global RNA‐chromatin interactome revealed by the GRID‐Seq technology (X. Li et al., 2017) and potential RNA–DNA–DNA triplex mediated lncRNA interactions predicted in the human genome (Jalali et al., 2017) confirm these findings, as also evaluated in detail on the example of the well studied lncRNAs‐MALAT1 and NEAT1. The fact that these interactions are predominantly observed in promoter regions strongly supports their regulatory role.

The deduced cross‐talk between the key epigenetic regulators with direct impact on the expression of protein‐coding genes provides a wealth of information on the cellular demands for sustenance under changing conditions. This study reveals different mechanisms of response and dynamics that provide cells with the tools to handle and adapt to both short‐term and long‐term changes by different, but interacting mechanisms. These mechanisms include: (a) ON/OFF mechanisms such as CpG island methylation in promoter regions, (b) the staged presence of activating and repressing histone marks that enable either stable expression or moderate upregulation or downregulation, and (c) the overlay of noncoding RNA regulation, that enables rapid and possibly transient DE to higher degrees. Further studies to obtain a more detailed understanding of how these regulatory mechanisms determine process behavior of cells and their ability to adapt to a variety of culture conditions will also increase our ability to control and manipulate gene expression towards more reliable process performance and outcome.

As an example, the pathway analysis over time of genes that are DE during stationary and decline phase indicates the struggle of cells to maintain homeostasis. These results might be used to understand the changes in product quality or productivity during late production process stages and indicate alleviatory feeding strategies, to ensure proper processing of the product. For instance, in the context of the cells, it makes sense to mobilize lipids under nutrient limitation and to initiate degradation of dispensable cellular components (as observed in the upregulation of lysosome and lipid metabolism), however, for protein production, lipids are essential as they are required for the generation of organelles, such as the endoplasmic reticulum and the Golgi, which are known to be bottlenecks of secretion. Likewise, the fact that “galactose metabolism” pops up as DE in the decline phase is critical as galactose is an important sugar required for proper glycoprocessing and thus the quality of the product.

For cell engineering approaches, the observed rapid response mechanism and control over gene expression levels exerted by lncRNAs also opens up the opportunity for completely new, so far unused tools for manipulating gene expression. Similar to microRNA (miRNA) engineering approaches, where the aim was to target the translation of multiple genes without burdening the overall protein production capacity of a cell, lncRNAs are no burden on the translational machinery and could be used to control gene transcription of target genes rather than their translation, thus intervening at an even earlier stage. While miRNAs can reduce translation of their target, engineering by lncRNAs could also be used to enhance transcription of individual genes, such as the product gene, an approach that has already been shown to work at the level of mRNA translation using lncRNAs (Takahashi et al., 2018). Speculatively, and excitingly, one could reach a level of understanding that allows control of entire phenotypes—which are determined by transcriptome patterns—by targeting multiple genes in a pathway without interfering with their genomic sequence or context by introducing lncRNAs designed to manipulate their expression patterns. This would be of interest particularly for controlling the precise expression level of a gene rather than turning it off or overexpressing it to a high level.

In conclusion, our report presents plausible mechanisms of regulation of gene expression in cells, from an ON/OFF switch, to mechanisms controlling constitutive gene expression, to such that determine changes in the level of gene expression in response to altered nutrient supply and waste accumulation as observed during a batch culture. A more detailed understanding of these mechanisms and cellular response to culture conditions will enable enhanced process control for bioproduction and innovative approaches for cell engineering and optimization.

## AVAILABILITY

The improved annotation is available for download at (http://denovo.cnag.cat/genomes/cgriseus/). Chromatin states, genome sequence, and DNA methylation data are available at http://cho‐epigenome.boku.ac.at/

## ACCESSION NUMBERS

DNA‐ and BS‐seq raw data: PRJEB9185; ChIP‐seq and RNA‐seq data: PRJEB9291.

## Supporting information

Supporting information

Supporting information

Supporting information
