# Supplementary material for: Epigenetic regulation of gene expression in Chinese Hamster Ovary cells in response to the changing environment of a batch culture
Source: Biotechnol Bioeng. 2019 Jan 4;116(3):677–92. doi: 10.1002/bit.26891 (PMC6492168; doi:10.1002/bit.26891)
Supplement: Supplementary file 1 — Supporting information [file BIT-116-677-s001.docx]

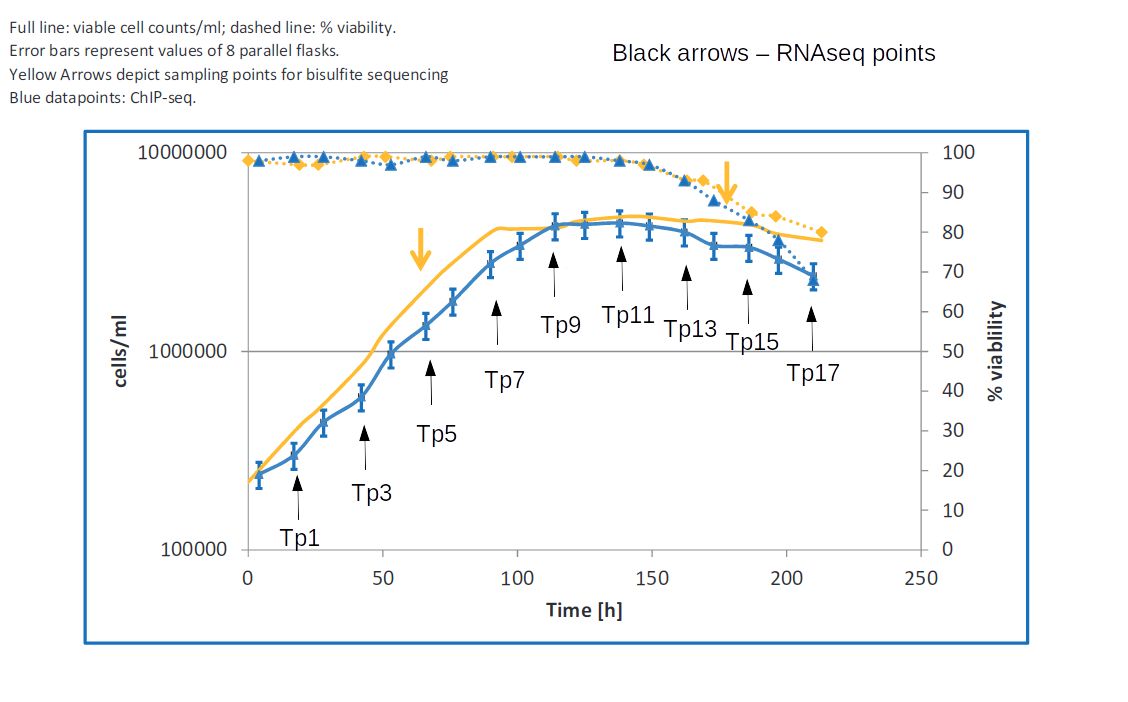


**Supplementary figure 1.** **Batch culture and sampling points.** ***Full line:*** *viable cell counts/ ml;* ***Dashed line:*** *% viability.* ***Error bars:*** *values of 8 parallel flasks.* ***Yellow arrows:*** *sampling points for bisulfite sequencing;* ***Black arrows:*** *sampling points for RNA-Seq;* ***Blue dots:*** *sampling marks for ChIP-Seq. (Figure modified from Feichtinger et. al. 2016)*

**
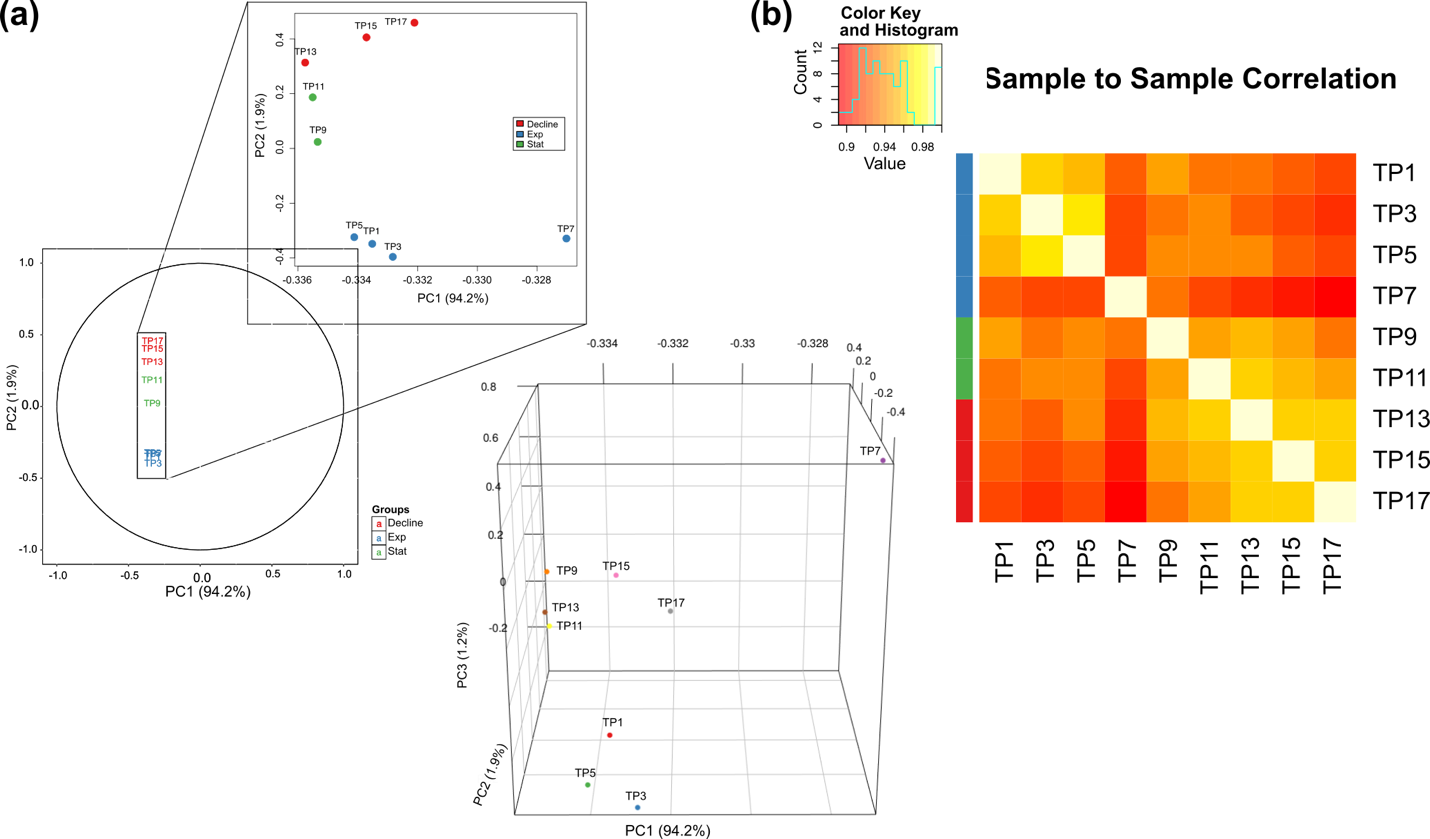
**

**Supplementary figure 2: (a) PCA plot of different RNA-Seq time points over batch culture.** *PC1 (94.2 % explained variability) vs PC2 (1.9% explained variability) shows overall similarity between samples across PC1 and clustering of samples over different growth phases during batch culture across PC2. Zoomed in view shows separation of samples within very small range across PC1. 3D plot showing the clustering of samples based on first three principal components*
**(b) Heatmap Spearman correlation between different RNAseq time points over batch culture.** *We observe a high correlation until middle exponential point (Tp5_exponential), Tp7 is transition between exponential and stationary phase and Tp9 is the beginning of Stationary phase. Samples correlate by cell culture phase, with a clear shift between exponential and stationary phase. This heatmap has been done with all expressed genes, coding and non-coding. Note: Samples are very similar, because scale in the correlation heatmap ranges within 0.88 to 1.*


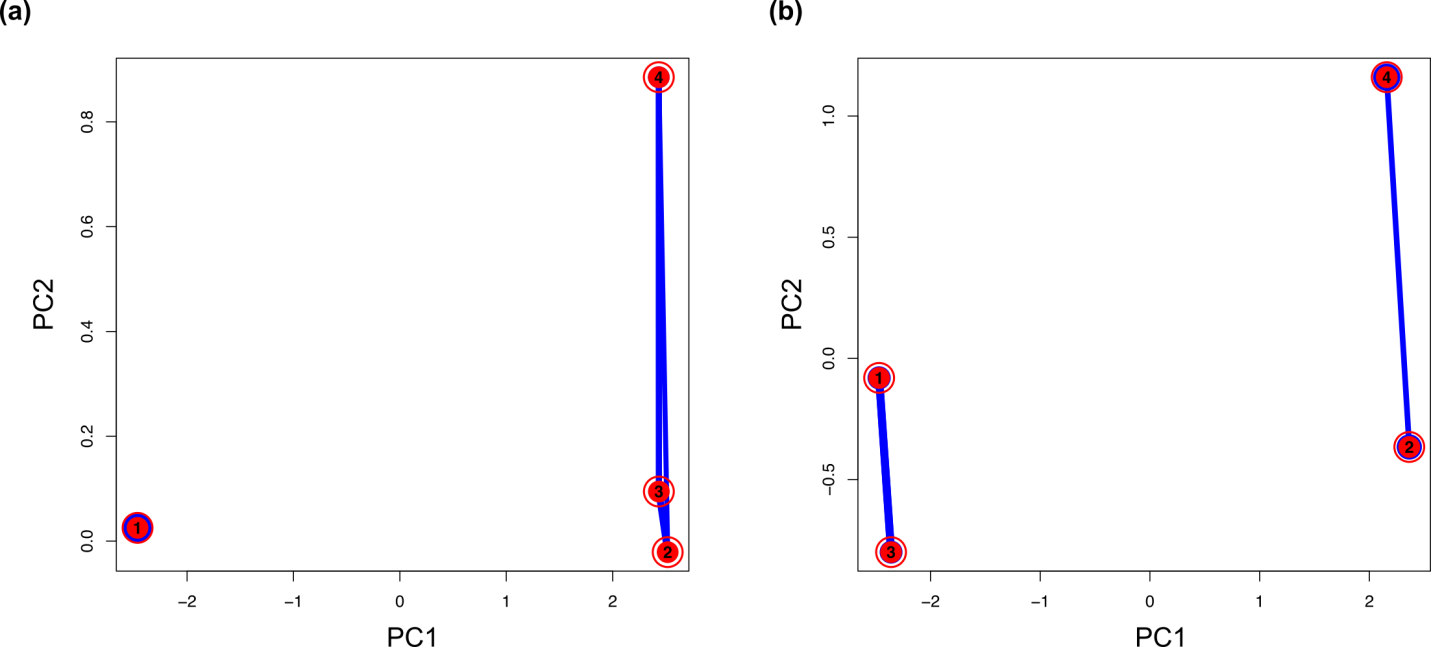


**Supplementary figure 3. Principal component analysis showing overlapping between clusters.** *Soft clustering allows genes to be part of several clusters. Similar overall trend seen in clusters can be confirmed by the overlap (depicted by lines) seen in figure a and b for coding genes and non-coding RNAs respectively.*


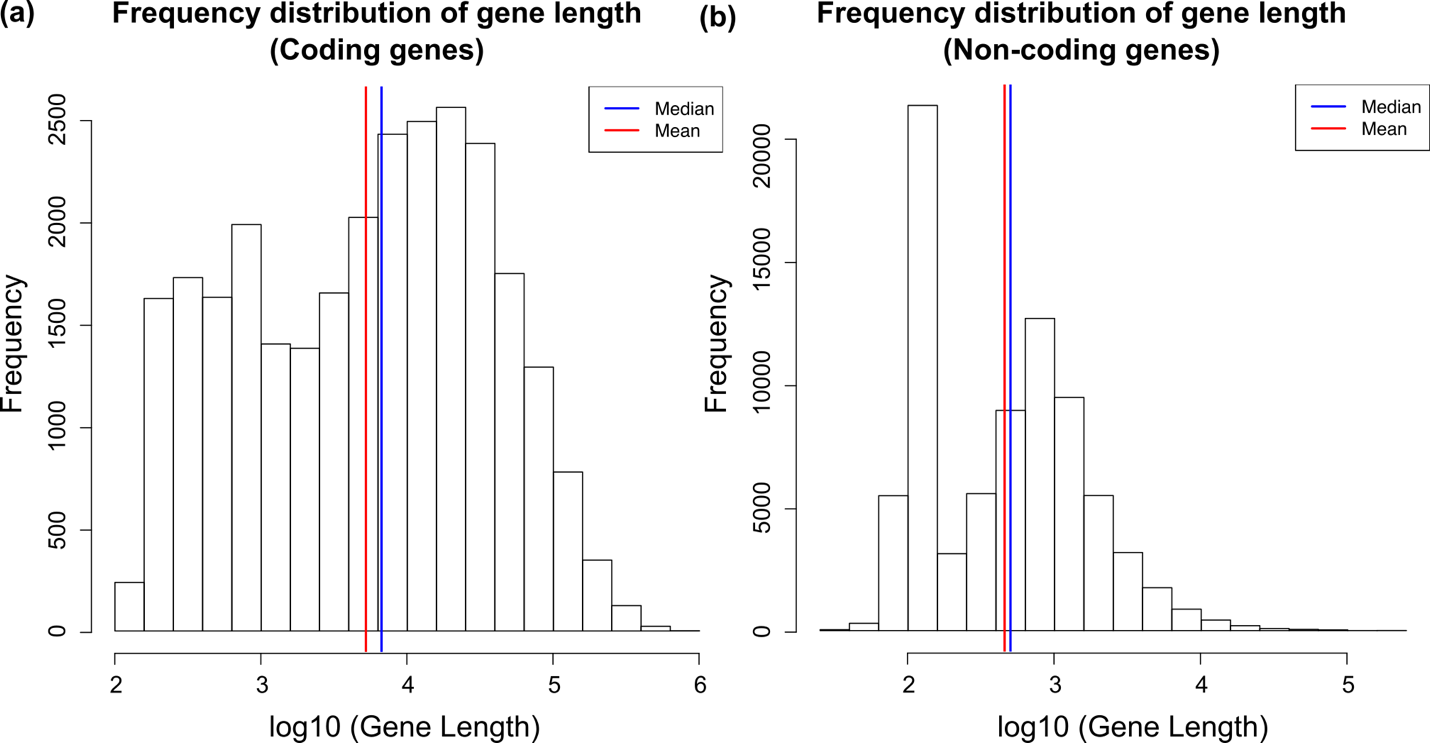


**Supplementary figure 4. Frequency distribution of length of (a) coding and (b) non-coding genes.** *X-axis represent log_10_ gene length values and Y-axis represent frequency of genes with corresponding gene length.*

**
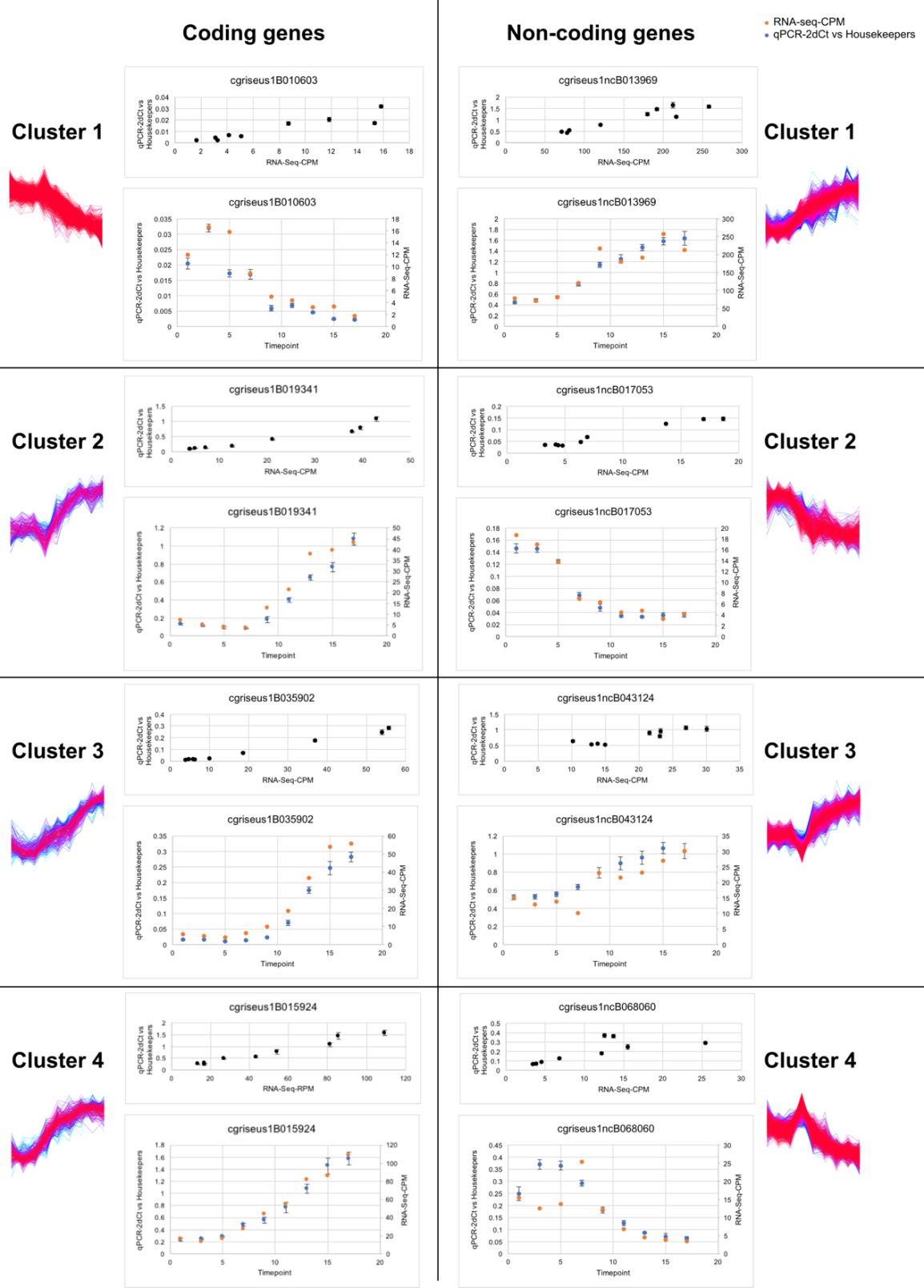
**

**Supplementary figure 5. qPCR validation plots.** *qPCR results for examples from all the four clusters of coding and non-coding transcribed regions. Upper plot for each example shows correlation between RNA-Seq and qPCR values and lower plot shows trend of expression values from RNA-Seq and qPCR across the batch. Overall trend for all the 4 clusters for coding and non-coding genes generated from RNA-seq matches the trend from qPCR. Line colors indicate membership values of each gene ranging from blue (for low membership) to red (for high membership)*


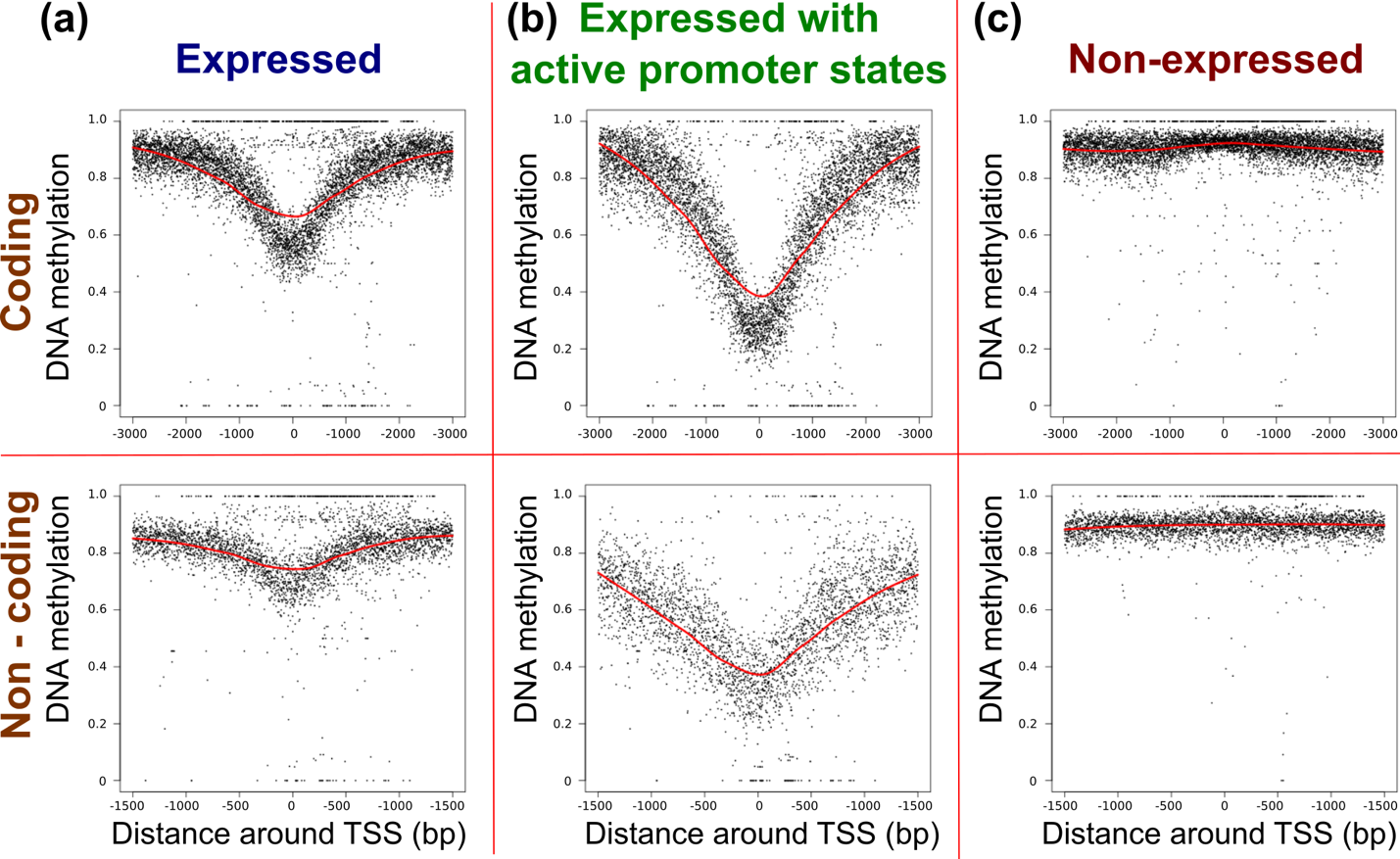


**Supplementary figure 6. Distinct patterns of DNA methylation around TSS for coding and non-coding genes.** *The figure shows clear a distinction of methylated and de-methylated regions around TSS of (a) expressed genes (b) expressed genes overlapping active promoter state and (c) non-expressed coding genes in the top panel and non-coding genes in the bottom panel. Increase in methylation levels while comparing the coding (top panel) with non-coding genes (bottom panel) and expressed (a, b) with non-expressed genes is also evident. The decrease in methylation levels for the expressed genes overlapping with active promoter states can also be appreciated while comparing (a) with (b), which highlights the fact that the promoter regions are highly de-methylated for expressed coding as well as non-coding genes.*


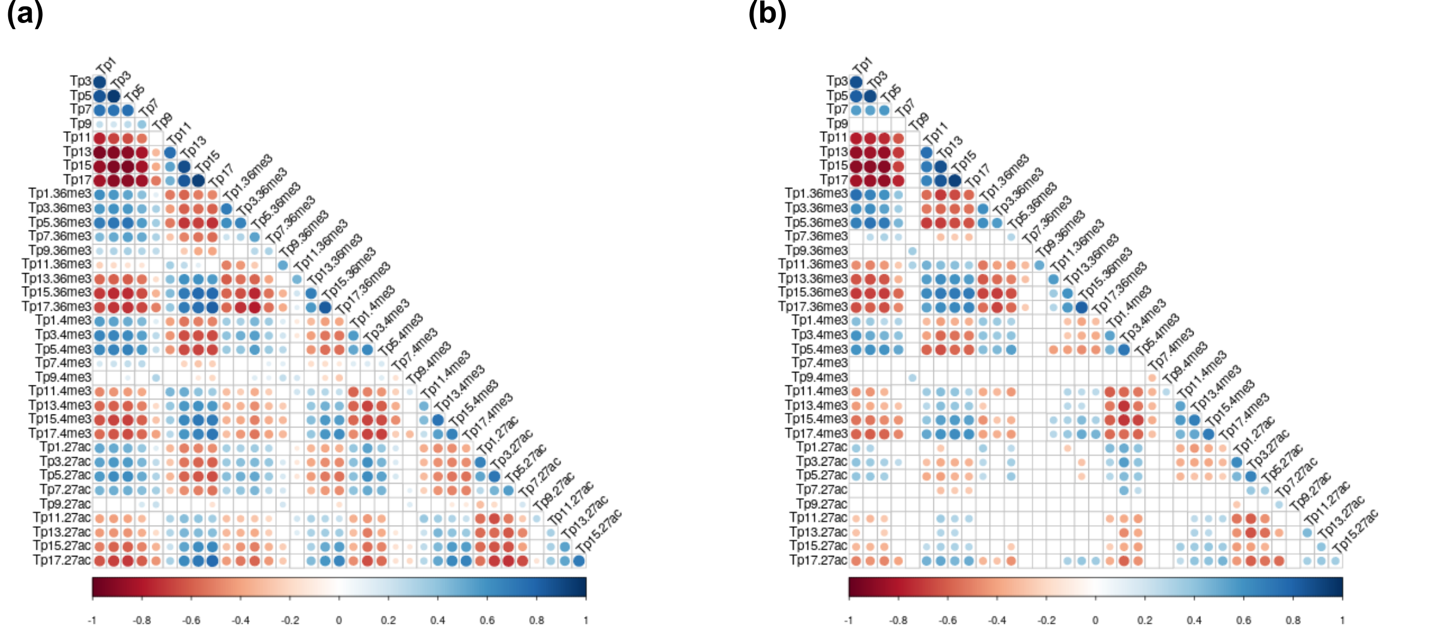


**Supplementary figure 7. Correlation between the changes in expression levels and changes in levels of histone modifications for DE genes. (a)** protein coding genes **(b)** lncRNAs

***Axis Labels:*** RNAseq VST normalized counts - *Tp1, Tp3, Tp5, Tp7, Tp9, Tp11, Tp13, Tp15, Tp17*

Normalized H3K36me3 CPM values within regions containing H3K36me3 peaks in gene body - *Tp1.36me3, Tp3.36me3, Tp5.36me3, Tp7.36me3, Tp9.36me3, Tp11.36me3, Tp13.36me3, Tp15.36me3 and Tp17.36me3*

Normalized H3K4me3 CPM values within regions around TSS (TSS+/-500 bp) containing H3K4me3 peaks and chromatin states 9 and 10 - *Tp1.4me3, Tp3.4me3, Tp5.4me3, Tp7.4me3, Tp9.4me3, Tp11.4me3, Tp4.36me3, Tp15.4me3 and Tp17.4me3*

Normalized H3K27ac CPM values within regions around TSS (TSS+/-500 bp) containing H3K27ac peaks and chromatin states 9 and 10 - *Tp1.27ac, Tp3.27ac, Tp5.27ac, Tp7.27ac, Tp9.27ac, Tp11.27ac, Tp4.27ac, Tp15.27ac and Tp17.27ac*

***Interpretation***

*Figure shows significant (pvalue < 0.01) Pearson correlation between expression levels from RNAseq, H3K36me3, H3K27ac and H3K4me3 marks from all samples detailed above. The color and size of the circle depends on the correlation levels between intersecting samples. As shown in legend, red color represents negative correlation and blue color represents positive correlation.*

*Figure 7a shows positive correlation between exponential RNAseq samples (Tp1, Tp3,Tp5,Tp7) with exponential H3K36me3 samples (Tp1.36me3, Tp3.36me3, Tp5.36me3 and Tp7.36me3) but negative correlation with decline H3K36me3 samples (Tp13.36me3, Tp15.36me3 and Tp17.36me3).*

*However, correlation between stationary RNAseq samples (Tp9 and Tp11) and stationary H3K36me3 samples (Tp9.36me3 and Tp11.36me3) is either not significant (pvalue > 0.01) or very low (light blue).*

*Figure 7b shows correlation between lncRNA samples for RNAseq and H3K36me3 is similar to coding genes. In contrast, correlation between RNAseq and H3K27ac or H3K4me3 is either not significant or very low.*


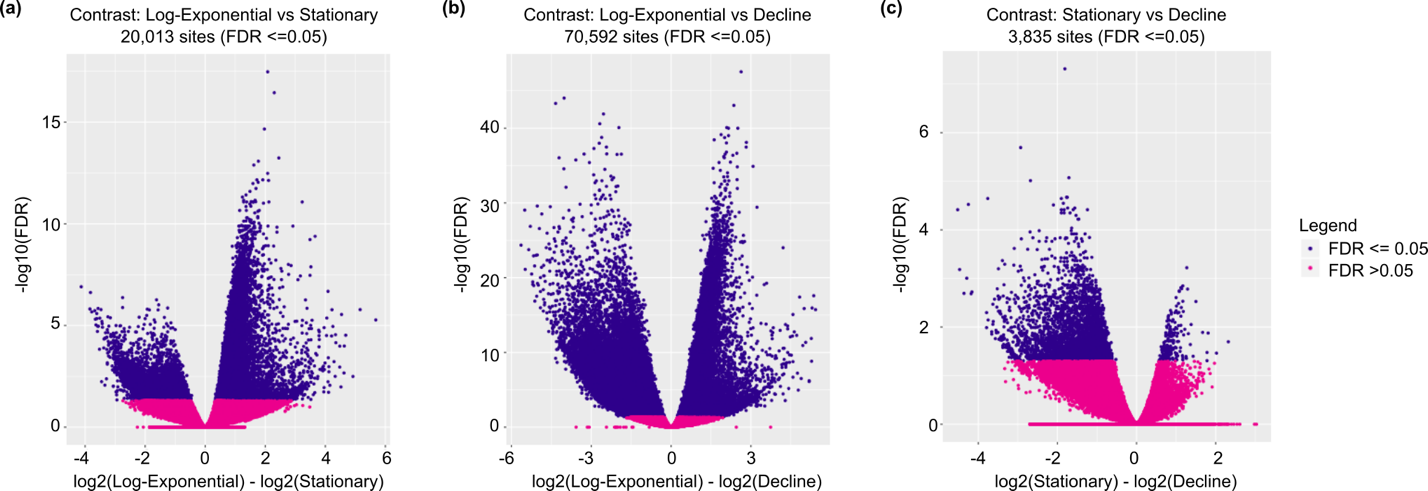


**Supplementary figure 8. Volcano plots showing significantly differentially acetylated sites within phase-wise comparisons (**as described in differential binding analysis of ChIPseq data from Feichinger *et al.***)**

***(a)*** ***Exponential (TP1, TP3, TP5 and TP7) vs Stationary (TP9 and TP11):*** *20,013 significantly differentially bound sites identified (FDR < 0.05) across the genome. Positive values correspond to differential bound sites in log exponential phase, negative values correspond to bound sites in stationary phase.* ***(b)*** ***Exponential vs Decline (TP13, TP15 and TP17):*** *70,592 significantly differentially bound sites identified (FDR < 0.05). Positive values correspond to differentially bound sites in log exponential phase and negative values to differentially bound sites in decline phase.* ***(c)*** ***Stationary vs Decline:*** *3,835 significantly differentially bound sites identified (FDR < 0.05). Sites identified as significantly differentially bound are shown in purple (FDR < = 0.05). Positive values correspond to differentially bound sites in stationary phase and negative values corresponds to differential bound sites in decline phase.*

*Number of significantly differential binding sites is found much higher between exponential and decline phase, where higher number of transcriptional changes occur (Figure 1, Figure 4) as compared to the sites found while comparing stationary versus decline phase.*

**Supplementary figure 9. Distribution range of expression levels for coding and non-coding transcribed regions.** *log10 FPKM values of median from all TPs in genes filtered for maximum expression level > 1 across all TPs (14,616 coding and 20,025 non-coding transcribed regions).*


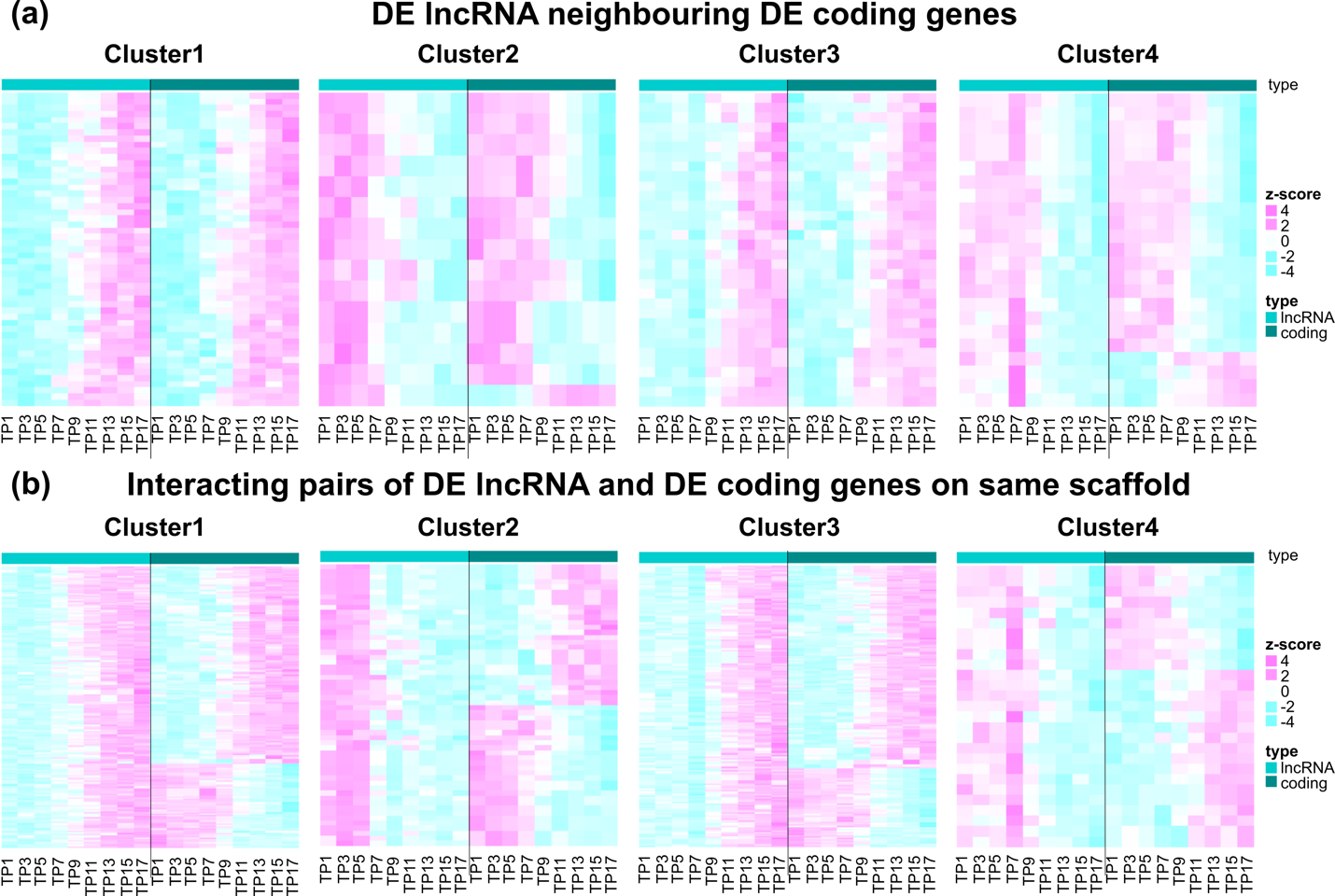


**SupplementaryFigure 10. Correlation of expression levels within the** *(a) neighboring lncRNA-coding gene pairs (b) interacting lncRNA-coding gene pairs (DE lncRNA, DE Coding genes). While it is evident to see such a strong pattern of differential expression of coding genes with the expression levels of neighboring or interacting lncRNA if the interactome is filtered for DE coding genes (as seen above), however it is interesting to observe similar pattern without filtering for DE genes (as in Figure 5 and 6a). This indicates probable causality of differential expression in coding genes by the neighboring lncRNA or interacting lncRNA with target sites nearby.*


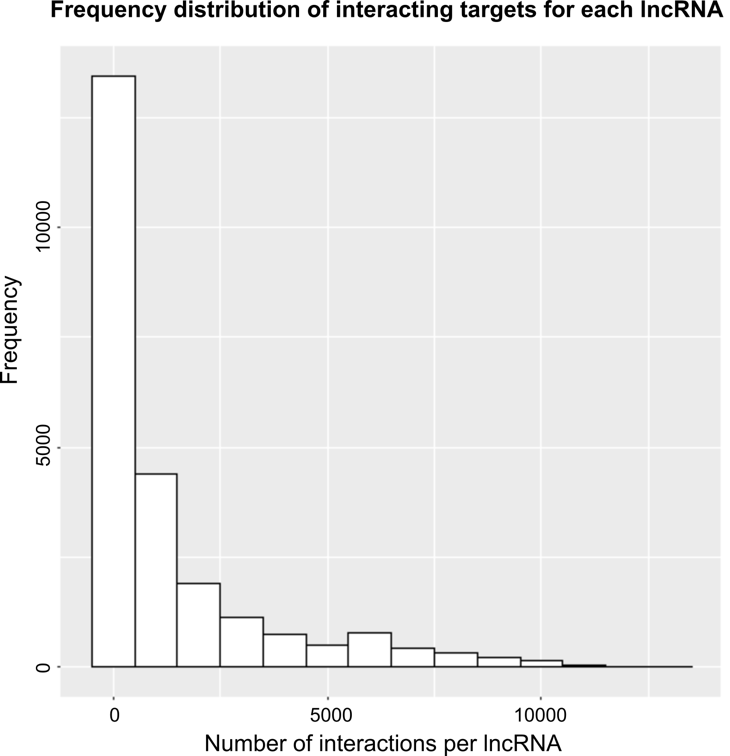


**SupplementaryFigure 11. Frequency distribution of interactions of each lncRNA with all coding genes.** *The figure reports number of coding genes with which each lncRNA interacts. As lncRNAs having more number of interactions would be connected to genes associated in more pathways, they can be considered to be better contributors for eliciting phenotypic changes.*


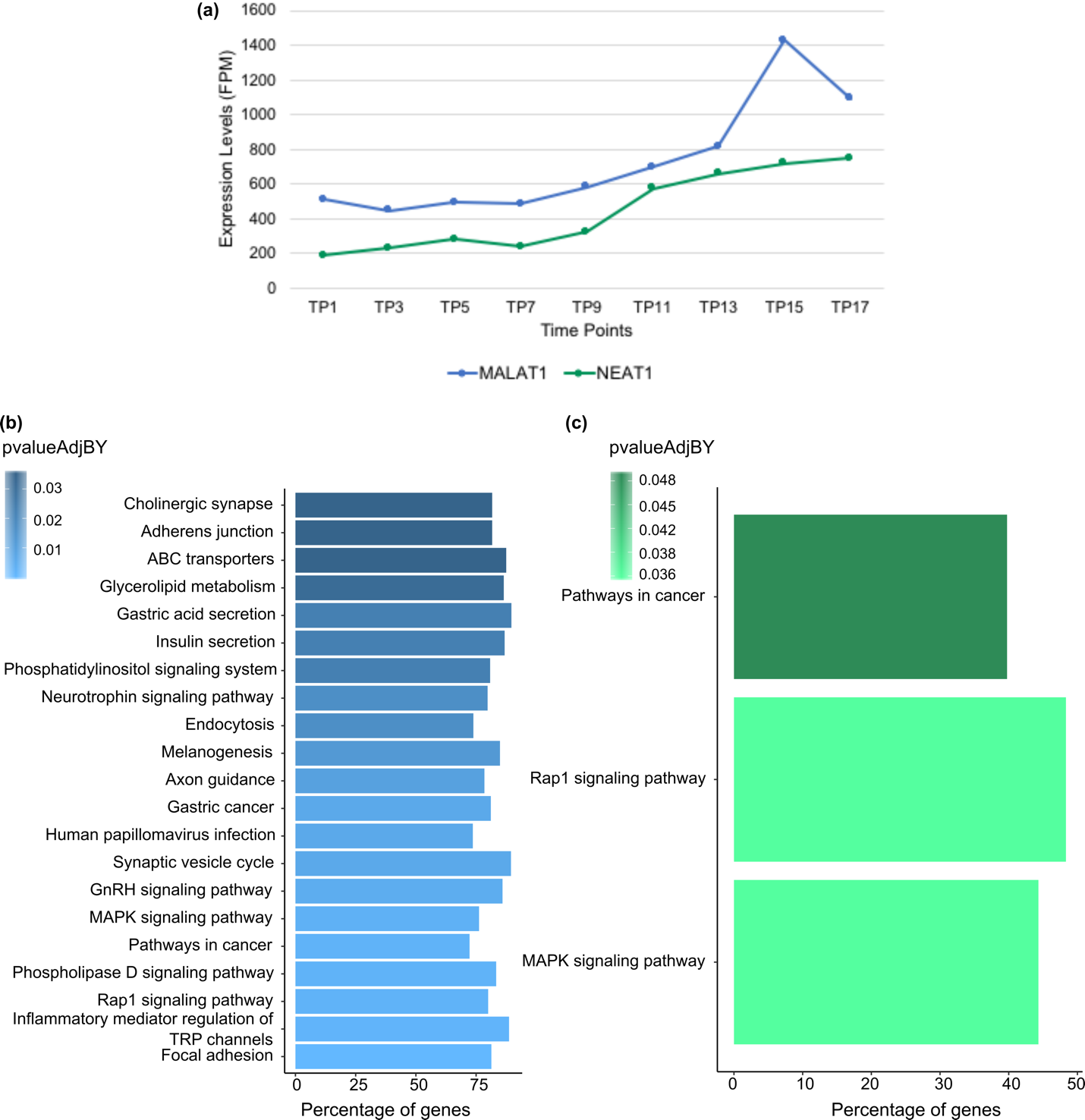


**SupplementaryFigure 12. Functional relevance of triplex mediated interaction list.** MALAT1 and NEAT1, considered the best characterized lncRNAs associated with tumorigenesis and nuclear speckles, are upregulated in the later phases of the batch culture. The interacting coding genes from the list of triplex mediated lncRNA-coding gene pairs corresponding to NEAT1 (cgriseus1ncB038456) and MALAT1 (cgriseus1ncB038456) are significantly enriched in KEGG pathways relevant for all the biological process associated with these lncRNAs. **(a)** Expression levels in FPM are plotted for MALAT1 (blue) and NEAT1 (green) at different time points in the batch culture. MALAT1 and NEAT1 fall in the cluster 3 and cluster 1 of significantly DE genes respectively. **(b) and (c)** report the KEGG pathways enriched in list of genes interacting with MALAT1 and NEAT1 respectively with length of bars representing percentage of genes found in the list amongst total expressed genes in our cell line associated with the pathway. Intensity of bar colors correspond to the adjusted p-values based on Benjamini Yekutieli method for enrichment of KEGG pathways with hypergeometric test.
