# Supplementary material for: Epigenetic regulation of gene expression in Chinese Hamster Ovary cells in response to the changing environment of a batch culture
Source: Biotechnol Bioeng. 2019 Jan 4;116(3):677–92. doi: 10.1002/bit.26891 (PMC6492168; doi:10.1002/bit.26891)
Supplement: Supplementary file 3 — Supporting information [file BIT-116-677-s003.docx]

## Results (short)

1. Mappings of C. griseus RNA-seq, Chinese hamster ESTs deposited in public databases and the human, rodent and rabbit proteins were combined with ab initio gene predictions to produce a set of consensus CDS models, which were then updated to include UTR sequences and annotate alternative spliceforms. This resulted in a preliminary set of 27,799 genes, whose 36,318 transcripts encode 34,775 unique protein products (~1.31 transcripts per coding gene). We were capable of functionally annotate 25,541 out of the 27,799 protein coding genes.
2. 78,873 non-coding genes and 80,973 non-coding transcripts have been annotated. 51,193 out of the 80,973 transcripts are claimed to be long non-coding RNAs, while 3218 out of the 29,780 small non coding RNA genes have been annotated as tRNAs by tRNAscan-SE. The other 26,562 genes are members of some of the non-coding families present in the RFAM database.
3. We studied the presence of activating chromatin marks and the length distribution of the 2,476 genes without a functional annotation and we finally them as: 1,528 non-coding genes and 947 protein-coding genes with unknown function. 19 out of the 947 protein-coding genes with unknown function are likely to be Chinese hamster orphan genes.

**New reference gene model built for coding and non-coding transcribed regions**

*Generation of Consensus Gene Models.* Gene annotation was performed by a combination of the Program to Assemble Spliced Alignments (PASA r2014-04-17) and Evidence Modeler (EVM r2012-06-25) (1) to obtain consensus coding sequence (CDS) models using three main sources of evidence: gene predictions, aligned transcripts, and aligned proteins.

*Transcript alignment.* Transcripts for assembly with PASA (PASA v-2.0.1) were obtained as follows: first, RNA-seq reads were aligned to the *Cricetulus griseus* assembly (Cgr1.0) with GEM and transcript models were generated using the standard Cufflinks (2) pipeline, resulting in 828,018 transcripts, which were then added to the PASA database. In addition, 1,462 *Cricetulus griseus* ESTs present in NCBI (July 10th, 2015) and the *Mus musculus* protein coding transcriptome downloaded from VEGA (Apr 9th, 2015) were also added to PASA using GMAP. All the above transcript alignments were then assembled by PASA, resulting in 413,622 assembled transcripts. The program TransDecoder was run with the obtained assemblies in order to detect coding regions, resulting in 67,142 predicted genes.

*Protein alignment.* We aligned the complete rodents and rabbit proteomes present in Uniprot (April 8, 2015), as well as the human proteins present in CCDS (April 7, 2015) to the genome with SPALN v2 (3) resulting in 1,573,279 CDS alignments.

*Ab initio gene predictions. Ab initio* gene predictions were performed on the reference genome masked for repeats which had been found with RepeatMasker (A.F.A. Smit, R. Hubley & P. Green RepeatMasker at [http://repeatmasker.org](http://repeatmasker.org/)) v4-0-5 using the rodentia library available in RepeatMasker. Also, transposable elements (TEs) were masked after getting BLAST (4) hits against the RepeatMasker database of proteins encoded by TEs. Low complexity repeats were left unmasked for this purpose. Three different gene prediction programs were used: GeneID (5), Augustus (6) and Genemark (7). GeneID *ab initio* gene predictions were obtained by running GeneID v1.4 with the pre-existing parameter file specific for *Homo sapiens* that has been previously used to accurately generate gene predictions in several different mammalian genomes (8, 9). For Augustus v3.0.2, we used the program’s pre-existing human parameter file. GeneMark-ES v2.3e gene predictions were obtained using its self-training mode. Hence, GeneID, Augustus and Genemark-ES were subsequently used to predict genes on the repeat-masked *Cricetulus griseus* assembly, made up of 28,751 scaffolds. The number of predicted gene models ranged from 33,968 with Augustus to 62,593 with Genemak-ES, while GeneID predicted 52,737.

*Gene predictions with hints.* GeneID, Augustus and Genemark-ET were also used to generate predictions incorporating intron evidence as produced from the RNA-seq data, by getting the junctions after running the GEMTools RNA-pipeline. Those junctions that mapped with *ab initio* GeneID or Augustus predictions or with protein mappings were taken as intron evidence. Running GeneID with introns resulted in a total set of 24,978 gene models, Augustus with introns resulted in 23,059 and Genemark-ET, in 86,042.

*Generation of consensus CDS models.* Transcript alignments, protein alignments, the models produced together with the previous annotation were combined into consensus CDS models using EvidenceModeler (EVM). EVM was run with five different sets of weights and the resulting consensus models with the best specificity and sensitivity as determined by intersection (BEDTools (10)) with the transcript mappings were chosen for the final annotation (SupplTable 1). These were then updated with UTRs and alternative exons through two rounds of PASA annotation updates. A final round of quality control was performed, fixing reading frames, intron phases and removing some transcripts that would be subject to nonsense-mediated decay (NMD). The resulting transcripts were clustered into genes using shared splice sites or significant sequence overlap as criteria for designation. Systematic identifiers with the prefix “cgriseus1B” were assigned to the genes, transcripts and protein products derived from them. Support by source of evidence at the gene and exon level was determined a posteriori using BEDTools intersect and multi-inter programs.

*Protein-coding functional annotation.* In order to assign a functional description to the structurally annotated genes we used Trinotate (<https://trinotate.github.io/>), that basically consists of running blastp and blastx (4) to compare protein products against the SwissProt and Uniref90 databases. Next, the program HMMER (11) was run to detect PFAM (12) domains. Finally, the outputs of these programs were combined into a database by using Trinotate sqlite, to get functional descriptions, domains and Gene Ontologies for each gene.

*Non-coding RNA annotation.* Non-coding RNAs (ncRNAs) were annotated by the following steps. First, the program *cmsearch* (v1.1), that comes with Infernal (13), was run with the RFAM database of RNA families (v12.0) (14). Also, tRNAscan-SE (v1.23) (15) was run to detect transfer RNA genes. For long non-coding RNAs (lncRNAs) we first selected PASA-assemblies that had not been included in the annotation of protein-coding genes, i.e. expressed genes that were not translated to protein. Those that were longer than 200bp and whose length was not covered to at least 80% by a small ncRNA were incorporated into the ncRNA annotation as lncRNAs. The resulting transcripts were clustered into genes using shared splice sites or significant sequence overlap as criteria. Systematic identifiers with the prefix “cgriseus1ncB” were assigned to the genes and transcripts derived from them.

*Final Annotation results:* Mappings of *C. griseus* RNA-seq, ESTs deposited in public databases along-with the human, rodent and rabbit proteins were combined with *ab initio* gene predictions to produce a set of consensus CDS models, which were then updated to include UTR sequences and to annotate alternative splice variants. This resulted in a preliminary set of 27,799 protein coding genes, whose 36,318 transcripts encode 34,775 unique protein products (~1.31 transcripts per coding gene) including functional annotation for 25,541 genes (SupplTable 2). Based on the presence of active chromatin marks (H3K27ac, H3K36me3 and H3K4me3) and the length distribution of the 2,476 genes without a functional annotation, we finally annotated them as: 1,528 non-coding transcribed regions and 947 protein-coding genes with unknown function. 19 of the 947 protein-coding genes with unknown function are likely to be Chinese hamster orphan genes.

In addition to the protein-coding genes, 78,873 non-coding transcribed regions encoding for 80,973 transcripts were annotated. Of these, 51,193 are claimed to be for long non-coding RNAs, while 3,218 out of the 29,780 small non-coding RNAs were annotated as tRNAs (15). The other 26,562 transcribed regions are members of the non-coding families present in the RFAM database (14). As expected, the results from differential study of the chromatin marks in the annotated genes showed (SupplTable 3a) a clear difference of presence of activating chromatin marks between the protein-coding genes with functional annotation and the non-coding genes. But surprisingly, the behavior of the genes without functional annotation was more similar to that of the non-coding genes, suggesting that in that set we could have some non-coding genes. Determining which of those genes were non-coding and which ones were real protein-coding was based on SupplFig. 1 and SupplTable 3b.

**
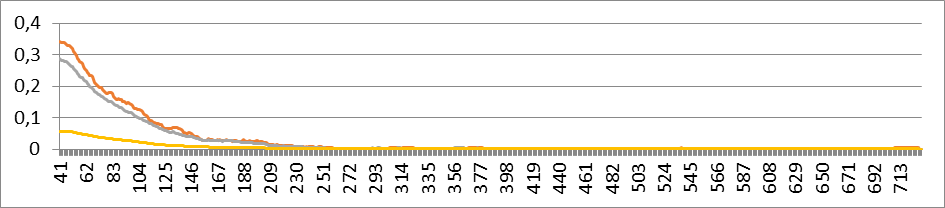
**

**Supplementary figure 1. Study of the length of the candidates to be orphan genes with respect to the length of the total set of genes.** *X-axis: length (bp); y-axis: relative presence of potential orphan genes in the total set of genes of length X+20bp (orange), X+40bp (grey), and all the genes of length equal or longer to X.*

**References:**

1. Haas,B.J., Salzberg,S.L., Zhu,W., Pertea,M., Allen,J.E., Orvis,J., White,O., Buell,C.R. and Wortman,J.R. (2008) Automated eukaryotic gene structure annotation using EVidenceModeler and the Program to Assemble Spliced Alignments. *Genome Biol.*, **9**, R7.

2. Trapnell,C., Williams,B.A., Pertea,G., Mortazavi,A., Kwan,G., van Baren,M.J., Salzberg,S.L., Wold,B.J. and Pachter,L. (2010) Transcript assembly and quantification by RNA-Seq reveals unannotated transcripts and isoform switching during cell differentiation. *Nat. Biotechnol.*, **28**, 511–515.

3. Iwata,H. and Gotoh,O. (2012) Benchmarking spliced alignment programs including Spaln2, an extended version of Spaln that incorporates additional species-specific features. *Nucleic Acids Res.*, **40**, e161–e161.

4. Altschul,S.F., Gish,W., Miller,W., Myers,E.W. and Lipman,D.J. (1990) Basic local alignment search tool. *J. Mol. Biol.*, **215**, 403–410.

5. Parra,G., Blanco,E. and Guigó,R. (2000) GeneID in Drosophila. *Genome Res.*, **10**, 511–515.

6. Stanke,M. and Waack,S. (2003) Gene prediction with a hidden Markov model and a new intron submodel. *Bioinforma. Oxf. Engl.*, **19 Suppl 2**, ii215-225.

7. Lomsadze,A., Ter-Hovhannisyan,V., Chernoff,Y.O. and Borodovsky,M. (2005) Gene identification in novel eukaryotic genomes by self-training algorithm. *Nucleic Acids Res.*, **33**, 6494–6506.

8. Abascal,F., Corvelo,A., Cruz,F., Villanueva-Cañas,J.L., Vlasova,A., Marcet-Houben,M., Martínez-Cruz,B., Cheng,J.Y., Prieto,P., Quesada,V., *et al.* (2016) Extreme genomic erosion after recurrent demographic bottlenecks in the highly endangered Iberian lynx. *Genome Biol.*, **17**, 251.

9. Mouse Genome Sequencing Consortium, Waterston,R.H., Lindblad-Toh,K., Birney,E., Rogers,J., Abril,J.F., Agarwal,P., Agarwala,R., Ainscough,R., Alexandersson,M., *et al.* (2002) Initial sequencing and comparative analysis of the mouse genome. *Nature*, **420**, 520–562.

10. Quinlan,A.R. and Hall,I.M. (2010) BEDTools: a flexible suite of utilities for comparing genomic features. *Bioinforma. Oxf. Engl.*, **26**, 841–842.

11. Finn,R.D., Clements,J. and Eddy,S.R. (2011) HMMER web server: interactive sequence similarity searching. *Nucleic Acids Res.*, **39**, W29-37.

12. Finn,R.D., Bateman,A., Clements,J., Coggill,P., Eberhardt,R.Y., Eddy,S.R., Heger,A., Hetherington,K., Holm,L., Mistry,J., *et al.* (2014) Pfam: the protein families database. *Nucleic Acids Res.*, **42**, D222-230.

13. Nawrocki,E.P. and Eddy,S.R. (2013) Infernal 1.1: 100-fold faster RNA homology searches. *Bioinforma. Oxf. Engl.*, **29**, 2933–2935.

14. Nawrocki,E.P., Burge,S.W., Bateman,A., Daub,J., Eberhardt,R.Y., Eddy,S.R., Floden,E.W., Gardner,P.P., Jones,T.A., Tate,J., *et al.* (2015) Rfam 12.0: updates to the RNA families database. *Nucleic Acids Res.*, **43**, D130-137.

15. Lowe,T.M. and Eddy,S.R. (1997) tRNAscan-SE: a program for improved detection of transfer RNA genes in genomic sequence. *Nucleic Acids Res.*, **25**, 955–964.
